# Translation and cross-cultural adaptation of the Noise Exposure Questionnaire (NEQ) to Brazilian Portuguese

**DOI:** 10.1590/2317-1782/20212022062en

**Published:** 2023-07-07

**Authors:** Letícia Campos de Oliveira, Clayton Henrique Rocha, Carla Gentile Matas, Karina Mary de Paiva, Renata Rodrigues Moreira, Alessandra Giannella Samelli

**Affiliations:** 1 Departamento de Fisioterapia, Fonoaudiologia e Terapia Ocupacional, Faculdade de Medicina - FMUSP, Universidade de São Paulo - São Paulo (SP), Brasil.; 2 Departamento de Fonoaudiologia, Universidade Federal de Santa Catarina - Florianópolis (SC), Brasil.; 3 Hospital Universitário - HU, Universidade de São Paulo - São Paulo (SP), Brasil.

**Keywords:** Translation, Hearing, Hearing Loss, Noise-Induced, Noise, Questionnaires

## Abstract

**Objective:**

To translate and cross-culturally adapt the Noise Exposure Questionnaire (NEQ) and 1-Minute Noise Screen (NEQ-S) instruments to Brazilian Portuguese.

**Methods:**

Procedures widely known in health research were used in the translation and cross-cultural adaptation process, comprising the following steps: initial translation, synthesis of translations, back-translation, expert committee, pretest, and content and layout validation. Altogether, 60 workers participated in the pretest by answering the questionnaires and then evaluating them in terms of understandability, layout, clarity, and writing. Reliability was verified with Cohen's kappa test, and the internal consistency was analyzed with Cronbach’s alpha coefficient.

**Results:**

The translated and adapted versions of NEQ and NEQ-S were similar in terms of general and referential meanings. However, some modifications and adaptations were made to adapt them to the Brazilian reality. The kappa test indicated moderate agreement and Cronbach’s alpha coefficient, substantial internal consistency.

**Conclusion:**

The translation and cross-cultural adaptation were carried out according to the methodology recommended in the national and international literature, performing the necessary equivalences to maintain the face and content validity with the original instrument. The availability of NEQ and NEQ-S in Brazilian Portuguese opens new fields of research to quantify yearly noise exposure more in-depth.

## INTRODUCTION

Studies increasingly address the knowledge about noise-related hearing loss, probably due to important research results involving guinea pigs^(1)^, demonstrating noise-induced cochlear synaptopathy^(2)^. In guinea pig studies, noise-exposure variables can be carefully controlled (frequency, intensity level, and exposure time), ensuring precise estimates of the association between noise exposure and hearing loss^(3)^. In human studies, dosimetry is the recommended technique in prospective assessments of these variables. However, retrospective estimates depend predominantly on self-reports of cumulative noise exposure, in which questionnaires are the indicated instruments to obtain such information^(2,3)^.

Approximately 27.7 million people aged 20 to 69 years in the United States are estimated to live with noise-induced hearing loss (NIHL)^(4)^. Moreover, NIHL is still the second most self-reported occupational disease, despite the regulations and interventions at the workplaces^(5,6)^.

The high prevalence of NIHL has been associated with increased industrialization, difficulties developing and implementing adequate public policies and preventive measures against noise, and difficulties related to information systems and data collection to generate consistent and comparable indicators^(7)^.

Hence, given the limited evidence on NIHL prevention and control and the high NIHL rates worldwide, further studies must be developed in this area, including the development of instruments to estimate occupational and non-occupational noise exposure, as gaps still exist. These include the unstandardized procedures to collect self-reported information, estimate the auditory risk (defining non-occupational risk factors), and establish validated instruments (accessing instruments or instructions for their use)^(2)^.

Standardizing instruments in the self-report process can minimize the effects of the subjective perception of risk - as there are different notions of the risk to which workers are exposed even when they have identical functions in common settings. These notions are based on practical knowledge, deductions, conversations with workmates, and information provided by the company^(2,8,9)^.

In this perspective, the task-based Noise Exposure Questionnaire (NEQ) was developed in detail to quantify people’s history of exposure to occupational and non-occupational noise. Also, the 1-Minute Noise Screen (NEQ-S) was developed to identify individuals at greater risk of developing NIHL^(10)^.

Thus, given the scarcity of such tools in Brazil, the objective of this research was to translate and cross-culturally adapt NEQ and NEQ-S to Brazilian Portuguese.

## METHODS

This study was approved by the institution’s Ethics Committee (no. 858/08), and the use of NEQ to this end was authorized by one of its authors.

### Description of the instrument

The original NEQ has 10 questions that estimate people’s yearly noise exposure. Their answer options vary - “Never; Every few months; Monthly; Weekly; Daily”; “8 or more; 4 hours up to 8 hours; 1 hour up to 4 hours; Less than 1 hour” (referring to various noise-exposure situations), as well as “Never; Sometimes; Always” (regarding the use of personal protective equipment). There is also the screening instrument, NEQ-S, which can be used to estimate people’s risk of developing NIHL. It has three questions with the following answer options: “Never; Every few months; Monthly; Weekly; Daily”, whose scores are respectively 0 to 4. Screening scores equal to or higher than 5 indicate a greater risk of developing NIHL.

### Procedures

The participants who agreed to participate in the research signed an informed consent form.

The study was conducted between March 2020 and December 2020. The translation and cross-cultural adaptation process followed procedures widely used in the health literature^(11,12)^.

**Figure 1:**
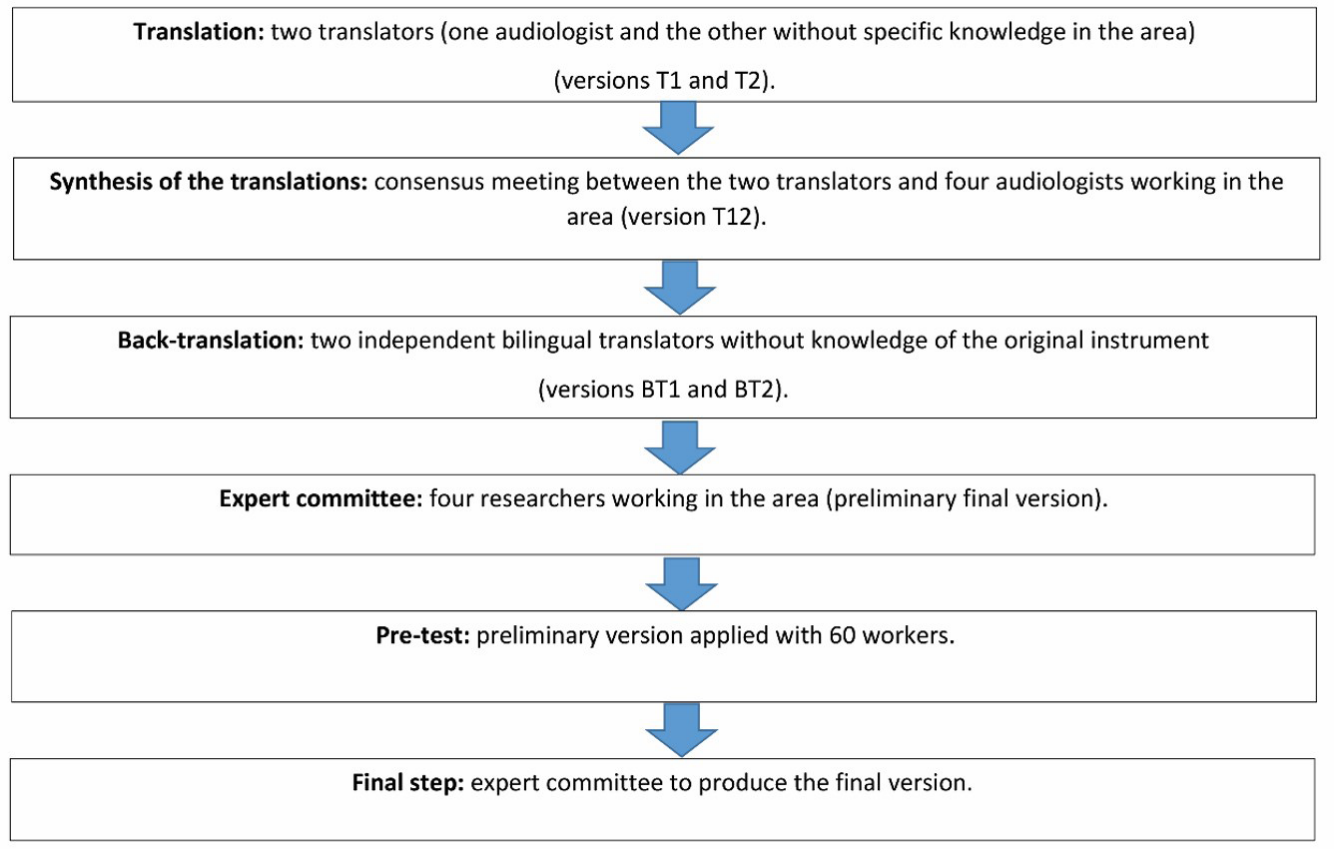
Flowchart - stages in the translation and cross-cultural adaptation of the Noise Exposure Questionnaire

The translation and cross-cultural adaptation of the instrument were conducted in the following stages: initial translation, synthesis of translations, back-translation, expert committee, pretest, and content and layout validation. These stages are described below^(11,12)^ (Figure 1):

- Translation and synthesis of the translations: two independent translations were made by two different translators fluent in English - one of them was an audiologist experienced in translation, and the other did not have specific knowledge in the area. Thus, versions T1 and T2 were created. These translations were then synthesized in a consensus meeting between the two authors and four audiologists’ researchers in the area. On this occasion, necessary reformulations were made, and the synthesized version of the initial translations was developed, which was named T12.
- Back-translation: T12 was back-translated from Portuguese into English by another two independent bilingual translators, who did not know the original instrument. Each back-translator produced a new version, named BT1 and BT2. This stage aimed to evaluate whether the content of the synthesized version was similar to that of the original instrument.
- Expert committee: the objective of this meeting was to produce the preliminary final version of the instrument to be used in the pretest. The translated versions were analyzed for semantic equivalence (vocabulary and grammar between the two languages, analyzing the reference meaning - denotation - and general meaning - connotation), idiomatic equivalence (proposing substitutes to idioms that are difficult to translate), experiential equivalence (proposing substitutes to experiences unparalleled in the country), and linguistic or conceptual equivalence (for words with different meanings in different cultures) with the original English questionnaires.
- Pretest: The preliminary final versions were used in a pretest with a group of workers to verify whether the questions were clear and easy to answer and identify possible comprehension problems in the questionnaires.
  • Final stage: After applying the pretest, all reports made throughout the process were submitted to the expert committee along with the preliminary translated version to verify whether the recommended stages had been followed and whether the reports reflected the process. Based on pretest results, small necessary final adjustments to the questionnaires were proposed.

To ensure that the pretest stage was adequate, the instrument was applied to a sample of individuals with similar characteristics to those for whom it had been designed^(11)^. The sample inclusion criteria were as follows: individuals older than 18 years; of both sexes; actively working at the university where the study was conducted; able to read and write; not having neurological, cognitive, and/or psychiatric disorders that might keep them from understanding the questions; having been submitted to audiometry at the institution’s audiology service within the previous year. The exclusion criteria were as follows: having any limiting factor that kept them from reading and filling out the questionnaires; having a conductive hearing loss.

Altogether, 62 workers participated in this stage. They worked in various areas (administration, kitchen, maintenance, metalworking, woodworking, general services, construction, and engineering) at the institution (public university) where the study was conducted, as recommended by the methodological reference used^(11)^. Two participants were excluded for not presenting the audiological examination in the stipulated time. Hence, the final sample had 60 participants.

Workers were contacted and invited to participate in the research. After agreeing and signing an informed consent form, participants answered the full questionnaires (including NEQ and NEQ-S). Then, they evaluated the questionnaires regarding comprehension, layout, clarity, and writing. They were also encouraged to suggest improvements when they found them appropriate.

### Statistical analysis

Reliability was verified with Cohen’s kappa test, and the internal consistency was analyzed with Cronbach’s alpha coefficient. Alpha (or kappa) values lower than 0.21 indicate weak; from 0.21 to 0.40, fair; from 0.41 to 0.60, moderate; from 0.61 to 0.80, substantial; and higher than 0.80, almost perfect internal consistency (or agreement)^(13)^.

## RESULTS

### Sample characterization

The 60 participants had a mean age of 44 years (SD = 12; Min: 22; Max: 65); 70% were males. Regarding educational attainment, most of them (47%) had a bachelor’s degree, followed by 43% with a high school degree; 10% of the participants had completed middle school.

As for the main characteristics of noise exposure, 52% were exposed to occupation noise and 48%, to non-occupational noise. In the audiometry, 68% of the assessed workers had normal hearing thresholds (up to 25 dB HL) in the left ear and 63%, in the right ear (Table 1).

**Table 1:** Sample characterization regarding noise exposure, hearing protection device use, and audiometry certificate

**Variable**	Exposure	N	**Percentage**
Occupational noise	No	29	48%
	Yes	31	52%
HPD	No	43	72%
	Yes	17	28%
Non-occupational noise	No	19	32%
	Yes	41	48%
RE audiometry			
Normal hearing*		38	63%
Hearing loss		22	37%
LE audiometry			
Normal hearing*		41	68%
Hearing loss		19	32%

* Hearing thresholds lower than or equal to 25 dB (HL) at all frequencies assessed in accordance with Regulation no. 19^(30)^)

**Caption:** HPD = Hearing protection device use; RE = Right ear; LE = Left ear

### Questionnaires

In general, the translated and back-translated NEQ and NEQ-S versions were similar regarding the general and reference meanings. Nonetheless, some changes (word and sentence adaptations, exclusions, and insertions) were made because some questions did not reflect Brazilian reality. Divergences were solved by consensus in the expert committee to make the questionnaires easier for the study population to understand.

The changes made were related to native language situations, colloquialisms, verbal phrases, and more than one possible translation.

The original versions, synthesis of the translations, synthesis of back-translations, and preliminary version (after expert committee analysis) are presented in Charts 1 and 2.

**Chart 1:** Questions in the Noise Exposure Questionnaire or *Questionário de Exposição ao Ruído* in the translation and cross-cultural adaptation process

QUESTIONS - NEQ	ORIGINAL VERSION IN ENGLISH	SUMMARY OF TRANSLATIONS INTO BRAZILIAN PORTUGUESE	SUMMARY OF BACK TRANSLATIONS INTO ENGLISH	COMMITTEE OF EXPERTS: EQUIVALENCE SEMANTIC, IDIOMATIC, EXPERIENCE AND LINGUISTIC - PRELIMINARY VERSION
INSTRUCTIONS	Please answer the following questions about yourself, **your hearing, and any noise you may have been around during** the past year. Write **an** answer **in the blank** [______] or **check** [ x ] the best answer **to** each question.	Por favor, responda as questões a seguir sobre si mesmo, sobre sua audição, e sobre qualquer ruído a que tenha sido exposto ao longo do último ano. Escreva sua resposta na lacuna [______] ou assinale [x ] a melhor resposta em cada questão.	Please answer the following questions about yourself, **about your hearing, and about any noise that you have been exposed over** the past year. Write **your** answer **on** [______] or **tick** [ ] the best answer **for** each question.	Por favor, responda as questões a seguir sobre si mesmo, sobre sua audição, e sobre qualquer ruído a que tenha sido exposto ao longo do último ano. Escreva sua resposta na lacuna [______] ou assinale [ x ] a melhor resposta em cada questão.
	Please answer these questions about any loud sounds. DURING THE PAST YEAR (12 months):	Por favor, responda estas perguntas referentes a qualquer som intenso.	Please answer these questions about any loud sounds. DURING THE PAST YEAR (12 months):	Por favor, responda estas perguntas referentes a qualquer som intenso.
		DURANTE O ÚLTIMO ANO (12 meses):		DURANTE O ÚLTIMO ANO (12 meses):
1	Outside of a paid job, how often did you use power tools, **chainsaws**, or other shop tools?	Sem ser em trabalho assalariado, com que frequência você usou ferramentas elétricas, serras elétricas, ou outras ferramentas de oficina?	Outside of a paid job, how often did you use power tools, **saws electrical**, or other tools?	Sem ser em trabalho assalariado, com que frequência você usou ferramentas elétricas, serras elétricas, ou outras ferramentas de oficina?
	⎕ Never ⎕ Every few months ⎕ Monthly ⎕ Weekly ⎕ Daily	⎕ Nunca ⎕ A cada 2 ou 3 meses ⎕ Mensalmente ⎕ Semanalmente ⎕ Diariamente	⎕ Never ⎕ Every few months ⎕ Monthly ⎕ Weekly ⎕ Daily	⎕ Nunca ⎕ A cada 2 ou 3 meses ⎕ Mensalmente ⎕ Semanalmente ⎕ Diariamente
	If you used power tools, on average, how many hours did each time/session last?	Se você usou ferramentas elétricas, por quanto tempo, em média, você as usou cada vez?	If you used power tools, on average, how many hours did each time/session last?	Se você usou ferramentas elétricas, por quanto tempo, em média, você as usou cada vez?
	⎕ 8 hours or more ⎕ 4 hours up to 8 hours ⎕ 1 hour up to 4 hours ⎕ Less than 1 hour	⎕ 8 horas ou mais ⎕ De 4 a 8 horas ⎕ De 1 a 4 horas ⎕ Menos de 1 hora	⎕ 8 hours or more ⎕ 4 hours up to 8 hours ⎕ 1 hour up to 4 hours ⎕ Less than 1 hour	⎕ 8 horas ou mais ⎕ De 4 a 8 horas ⎕ De 1 a 4 horas ⎕ Menos de 1 hora
	If you used power tools, how often did you wear earplugs or earmuffs during this activity?	Se você usou ferramentas elétricas, com que frequência você usou protetor auditivo **(tampão de ouvido ou abafador)** durante a atividade?	If you used power tools, how often did you wear earplugs or earmuffs during this activity?	Se você usou ferramentas elétricas, com que frequência você usou protetor auditivo **(tampão de ouvido ou abafador)** durante a atividade?
	⎕ Never ⎕ Sometimes ⎕ Always	⎕ Nunca ⎕ Às vezes ⎕ Sempre	⎕ Never ⎕ Sometimes ⎕ Always	⎕ Nunca ⎕ Às vezes ⎕ Sempre
2	Outside of a paid job, how often did you drive heavy **equipment or use loud** machinery (such as tractors, trucks, **or farming or lawn equipment like mowers/leaf blowers)**?	Sem ser em trabalho assalariado, com que frequência você dirigiu veículos pesados ou operou maquinários ruidosos (tais como trator, caminhão, maquinário de fazenda ou de quintal como cortador de grama ou soprador / aspirador de folhas)?	Outside of a paid job, how often did you drive heavy **vehicles or operated noisy** machinery (such as tractor, truck, **farm or yard machinery such as lawn mower or leaf blower/ vacuum cleaner)**?	Sem ser em trabalho assalariado, com que frequência você dirigiu veículos pesados ou operou maquinários ruidosos (tais como trator, caminhão, maquinário de fazenda ou de quintal como cortador de grama ou soprador / aspirador de folhas)?
	⎕ Never ⎕ Every few months ⎕ Monthly ⎕ Weekly ⎕ Daily	⎕ Nunca ⎕ A cada 2 ou 3 meses ⎕ Mensalmente ⎕ Semanalmente ⎕ Diariamente	⎕ Never ⎕ Every few months ⎕ Monthly ⎕ Weekly ⎕ Daily	⎕ Nunca ⎕ A cada 2 ou 3 meses ⎕ Mensalmente ⎕ Semanalmente ⎕ Diariamente
	If you **drove/used loud machinery, on average, how many hours did each time/session last**?	Se você dirigiu/operou maquinário ruidoso, por quanto tempo, em média, você o usou cada vez?	If you **have drove/operated noisy machinery, how long, on average, have you used it each time**?	Se você dirigiu/operou maquinário ruidoso, por quanto tempo, em média, você o usou cada vez?
	⎕ 8 hours or more ⎕ 4 hours up to 8 hours ⎕ 1 hour up to 4 hours ⎕ Less than 1 hour	⎕ 8 horas ou mais ⎕ De 4 a 8 horas ⎕ De 1 a 4 horas ⎕ Menos de 1 hora	⎕ 8 hours or more ⎕ 4 to 8 hours ⎕ 1 to 4 hours ⎕ Less than 1 hour	⎕ 8 horas ou mais ⎕ De 4 a 8 horas ⎕ De 1 a 4 horas ⎕ Menos de 1 hora
		Se você dirigiu/operou maquinário ruidoso, com que frequência você usou protetor auditivo (tampão de ouvido ou abafador) durante a atividade?		Se você dirigiu/operou maquinário ruidoso, com que frequência você usou protetor auditivo (tampão de ouvido ou abafador) durante a atividade?
	If you **drove/used machinery, how often did you wear earplugs or earmuffs during this activity**?	⎕ Nunca ⎕ Às vezes ⎕ Sempre	If you **drove/operated noisy machinery, how often did you use hearing protection equipment (earplugs or earmuffs) during the activity**?	⎕ Nunca ⎕ Às vezes ⎕ Sempre
	⎕ Never ⎕ Sometimes ⎕ Always		⎕ Never ⎕ Sometimes ⎕ Always	
3	How often did you attend car/truck races, commercial/**high schoo**l sporting events, music **concerts/dances** or any other events with amplified **public announcement (PA)/music systems**?	Com que frequência você foi a corridas de carros/caminhões, eventos esportivos comerciais/escolares, concertos/*shows* de música/festas ou quaisquer outros eventos com sistema de som amplificado usado para fazer anúncios ou tocar músicas?	How often did you attend **to** car/truck races, commercial/**school/religious** sporting events, concerts/**parties** or any other events with an amplified **sound system used to advertise or to play music**?	Com que frequência você foi a corridas de carro/caminhões, eventos esportivos comerciais/escolares/**religiosos**, concertos/*shows* de música/festas ou quaisquer outros eventos com sistema de som amplificado usado para fazer anúncios ou tocar músicas?
	⎕ Never ⎕ Every few months ⎕ Monthly ⎕ Weekly ⎕ Daily	⎕ Nunca ⎕ A cada 2 ou 3 meses ⎕ Mensalmente ⎕ Semanalmente ⎕ Diariamente	⎕ Never ⎕ Every few months ⎕ Monthly ⎕ Weekly ⎕ Daily	⎕ Nunca ⎕ A cada 2 ou 3 meses ⎕ Mensalmente ⎕ Semanalmente ⎕ Diariamente
	If you attended these events, on average, how many hours did each time/session last?	Se você esteve em tais eventos, quanto tempo, em média, cada um durou?	If you have been at such events, how long, on average, did each last?	Se você esteve em tais eventos, quanto tempo, em média, cada um durou?
	⎕ 8 hours or more ⎕ 4 hours up to 8 hours ⎕ 1 hour up to 4 hours ⎕ Less than 1 hour	⎕ 8 horas ou mais ⎕ De 4 a 8 horas ⎕ De 1 a 4 horas ⎕ Menos de 1 hora	⎕ 8 hours or more ⎕ 4 to 8 hours ⎕ 1 to 4 hours ⎕ Less than 1hour	⎕ 8 horas ou mais ⎕ De 4 a 8 horas ⎕ De 1 a 4 horas ⎕ Menos de 1 hora
	If you attended these events, how often did you wear earplugs or earmuffs during this activity?	Se você esteve em tais eventos, com que frequência você usou protetor auditivo (tampão de ouvido ou abafador) durante a atividade?	If you have been to such events, how often did you wear hearing protection equipment (earplugs or earmuffs) during the activity?	Se você esteve em tais eventos, com que frequência você usou protetor auditivo (tampão de ouvido ou abafador) durante a atividade?
	⎕ Never ⎕ Sometimes ⎕ Always	⎕ Nunca ⎕ Às vezes ⎕ Sempre	⎕ Never ⎕ Sometimes ⎕ Always	
				⎕ Nunca ⎕ Às vezes ⎕ Sempre
4	How often did you **ride**/operate motorized vehicles such as motorcycles, jet skis, speed boats, **snowmobiles, or four-wheelers**?	Com que frequência você dirigiu/operou veículos motorizados, tais como motocicletas, jet-skis, lanchas ou **quadriciclos**?	How often did you **drive**/operate motorized vehicles, such as motorcycles, jet skis, speed boats **or quads**?	Com que frequência você dirigiu/operou veículos motorizados, tais como motocicletas, jet-skis, lanchas ou quadriciclos**/buggy/Kart**?
	⎕ Never ⎕ Every few months ⎕ Monthly ⎕ Weekly ⎕ Daily	⎕ Nunca ⎕ A cada 2 ou 3 meses ⎕ Mensalmente ⎕ Semanalmente ⎕ Diariamente	⎕ Never ⎕ Every few months ⎕ Monthly ⎕ Weekly ⎕ Daily	⎕ Nunca ⎕ A cada 2 ou 3 meses ⎕ Mensalmente ⎕ Semanalmente ⎕ Diariamente
	If you **rode motorized** vehicles, **on average, how many hours did each time/session last**?	Se você dirigiu veículos motorizados, por quanto tempo, em média, você o usou cada vez?	If you **have driven motor** vehicles, **how long, on average, have you used it each time**?	Se você dirigiu veículos motorizados, por quanto tempo, em média, você o usou cada vez?
	⎕ 8 hours or more ⎕ 4 hours up to 8 hours ⎕ 1 hour up to 4 hours ⎕ Less than 1 hour	⎕ 8 horas ou mais ⎕ De 4 a 8 horas ⎕ De 1 a 4 horas ⎕ Menos de 1 hora	⎕ 8 hours or more ⎕ 4 to 8 hours ⎕ 1 to 4 hours ⎕ Less than 1 hour	⎕ 8 horas ou mais ⎕ De 4 a 8 horas ⎕ De 1 a 4 horas ⎕ Menos de 1 hora
	If you **rode motorized** vehicles, how often **did you wear earplugs or earmuffs during this** activity?	Se você dirigiu veículos motorizados, com que frequência você usou protetor auditivo (tampão de ouvido ou abafador) durante a atividade?	If you **have driven motorized** vehicles, how often **have you used hearing protection equipment (earplugs or earmuffs) during** activity?	Se você dirigiu veículos motorizados, com que frequência você usou protetor auditivo (tampão de ouvido ou abafador) durante a atividade?
	⎕ Never ⎕ Sometimes ⎕ Always	⎕ Nunca ⎕ Às vezes ⎕ Sempre	⎕ Never ⎕ Sometimes ⎕ Always	⎕ Nunca ⎕ Às vezes ⎕ Sempre
5	How often did you ride in or pilot small aircraft/private airplanes?	Com que frequência você voou em, ou pilotou, uma aeronave de pequeno porte/avião particular?	How often did you ride in or pilot small aircraft/private airplanes?	Com que frequência você voou em, ou pilotou, uma aeronave de pequeno porte/avião particular?
	⎕ Never ⎕ Every few months ⎕ Monthly ⎕ Weekly ⎕ Daily	⎕ Nunca ⎕ A cada 2 ou 3 meses ⎕ Mensalmente ⎕ Semanalmente ⎕ Diariamente	⎕ Never ⎕ Every few months ⎕ Monthly ⎕ Weekly ⎕ Daily	⎕ Nunca ⎕ A cada 2 ou 3 meses ⎕ Mensalmente ⎕ Semanalmente ⎕ Diariamente
	If you flew airplanes, on average, how many hours did each time/session last?	Se você voou em aeronaves, quanto tempo, em média, durou cada voo?	If you flew airplanes, on average, how many hours did each time/session last?	Se você voou em aeronaves, quanto tempo, em média, durou cada voo?
	⎕ 8 hours or more ⎕ 4 hours up to 8 hours ⎕ 1 hour up to 4 hours ⎕ Less than 1 hour	⎕ 8 horas ou mais ⎕ De 4 a 8 horas ⎕ De 1 a 4 horas ⎕ Menos de 1 hora	⎕ 8 hours or more ⎕ 4 hours up to 8 hours ⎕ 1 hour up to 4 hours ⎕ Less than 1 hour	⎕ 8 horas ou mais ⎕ De 4 a 8 horas ⎕ De 1 a 4 horas ⎕ Menos de 1 hora
	If you flew airplanes, how often did you wear earplugs or earmuffs during this activity?	Se você voou em aeronaves, com que frequência você usou protetor auditivo (tampão de ouvido ou abafador) durante a atividade?	If you flew airplanes, how often did you wear earplugs or earmuffs during this activity?	Se você voou em aeronaves, com que frequência você usou protetor auditivo (tampão de ouvido ou abafador) durante a atividade?
	⎕ Never ⎕ Sometimes ⎕ Always	⎕ Nunca ⎕ Às vezes ⎕ Sempre	⎕ Never ⎕ Sometimes ⎕ Always	⎕ Nunca ⎕ Às vezes ⎕ Sempre
6	How often **were you around or did you** shoot firearms such as rifles, pistols, shotguns, etc.?	Com que frequência você esteve próximo a, ou disparou, armas de fogo tais como rifles, pistolas, espingardas etc.?	How often **have you been close to, or** shoot firearms such as rifles, pistols, shotguns, etc.?	Com que frequência você esteve próximo a, ou atirou, armas de fogo tais como rifles, pistolas, espingardas etc.?
	⎕ Never ⎕ Every few months ⎕ Monthly ⎕ Weekly ⎕ Daily	⎕ Nunca ⎕ A cada 2 ou 3 meses ⎕ Mensalmente ⎕ Semanalmente ⎕ Diariamente	⎕ Never ⎕ Every few months ⎕ Monthly ⎕ Weekly ⎕ Daily	⎕ Nunca ⎕ A cada 2 ou 3 meses ⎕ Mensalmente ⎕ Semanalmente ⎕ Diariamente
	If you were **around/shot** firearms, **on average, how many shots** did you fire each **time/session**?	Se você esteve próximo a/disparou armas de fogo, quanto disparos, em média, você fez ou presenciou a cada vez?	If you were **close to/ shoot** firearms, **how many shots, on average**, did you fire each **time**?	Se você esteve próximo a/disparou armas de fogo, quanto disparos, em média, você fez ou presenciou a cada vez? _________ tiros de espingarda/rifle por vez. _________ tiros de pistola por vez.
	_________ shotgun/rifle shots **per session** _________ pistol shots **per session**	_________ tiros de espingarda/rifle por vez. _________ tiros de pistola por vez.	_____ shotgun/rifle shots **at a time**. _______ pistol shots **at a time.**	Se você esteve próximo a/disparou armas de fogo, com que frequência você usou protetor auditivo (tampão de ouvido ou abafador) durante os disparos?
	If you were **around/shot** firearms, how often did you wear earplugs or earmuffs **while shooting**?	Se você esteve próximo a/disparou armas de fogo, com que frequência você usou protetor auditivo (tampão de ouvido ou abafador) durante os disparos?	If you were close **to/shot** firearms, how often did you wear earplugs or earmuffs **during the shots**?	⎕ Nunca ⎕ Às vezes ⎕ Sempre
	⎕ Never ⎕ Sometimes ⎕ Always	⎕ Nunca ⎕ Às vezes ⎕ Sempre	⎕ Never ⎕ Sometimes ⎕ Always	
7	How often did you play a musical instrument?	Com que frequência você tocou um instrumento musical?	How often did you play a musical instrument?	Com que frequência você tocou um instrumento musical?
	⎕ Never ⎕ Every few months ⎕ Monthly ⎕ Weekly ⎕ Daily	⎕ Nunca ⎕ A cada 2 ou 3 meses ⎕ Mensalmente ⎕ Semanalmente ⎕ Diariamente	⎕ Never ⎕ Every few months ⎕ Monthly ⎕ Weekly ⎕ Daily	⎕ Nunca ⎕ A cada 2 ou 3 meses ⎕ Mensalmente ⎕ Semanalmente ⎕ Diariamente
	If you played, please tell us what musical instrument: _________________________	Se você tocou, por favor, nos conte qual instrumento: _________________________	If you played, please tell us which instrument: _________________________	Se você tocou, por favor, nos conte qual instrumento:
	If you played a musical instrument, **on average, how many hours did each time/session last**?	Se você tocou um instrumento musical, por quanto tempo, em média, você o tocou cada vez?	If you played a musical instrument **how long, on average, did you play it each time**?	Se você tocou um instrumento musical, por quanto tempo, em média, você o tocou cada vez?
	⎕ 8 hours or more ⎕ 4 hours up to 8 hours ⎕ 1 hour up to 4 hours ⎕ Less than 1 hour	⎕ 8 horas ou mais ⎕ De 4 a 8 horas ⎕ De 1 a 4 horas ⎕ Menos de 1 hora	⎕ 8 hours or more ⎕ 4 to 8 hours ⎕ 1 to 4 hours ⎕ Less than 1 hour	⎕ 8 horas ou mais ⎕ De 4 a 8 horas ⎕ De 1 a 4 horas ⎕ Menos de 1 hora
	If you played a musical instrument, how often did you wear earplugs or earmuffs while playing?	Se você tocou um instrumento musical, com que frequência você usou protetor auditivo (tampão de ouvido ou abafador) enquanto tocava?	If you played a musical instrument, how often did you wear earplugs or earmuffs while playing?	Se você tocou um instrumento musical, com que frequência você usou protetor auditivo (tampão de ouvido ou abafador) enquanto tocava?
	⎕ Never ⎕ Sometimes ⎕ Always	⎕ Nunca ⎕ Às vezes ⎕ Sempre	⎕ Never ⎕ Sometimes ⎕ Always	⎕ Nunca ⎕ Às vezes ⎕ Sempre
8	How often did you listen to music, radio programs, etc. **using personal headsets or earphones**?	Com que frequência você ouviu música, programas de rádio etc., usando fones de ouvido ou headset individuais?	How often did you listen to music, radio programs, etc., **wearing earphones or headsets**?	Com que frequência você ouviu música, programas de rádio etc., usando fones de ouvido ou *headset* individuais?
	⎕ Never ⎕ Every few months ⎕ Monthly ⎕ Weekly ⎕ Daily	⎕ Nunca ⎕ A cada 2 ou 3 meses ⎕ Mensalmente ⎕ Semanalmente ⎕ Diariamente	⎕ Never ⎕ Every few months ⎕ Monthly ⎕ Weekly ⎕ Daily	⎕ Nunca ⎕ A cada 2 ou 3 meses ⎕ Mensalmente ⎕ Semanalmente ⎕ Diariamente
	If you listened through earphones, **on average, how many hours did each time/session last**?	Se você ouviu em fones de ouvido, por quanto tempo, em média, você ouviu cada vez?	If you listened through earphones, **how long, on average, did you use each time**?	Se você ouviu em fones de ouvido, por quanto tempo, em média, você ouviu cada vez?
	⎕ 8 hours or more ⎕ 4 hours up to 8 hours ⎕ 1 hour up to 4 hours ⎕ Less than 1 hour	⎕ 8 horas ou mais ⎕ De 4 a 8 horas ⎕ De 1 a 4 horas ⎕ Menos de 1 hora	⎕ 8 hours or more ⎕ 4 to 8 hours ⎕ 1 to 4 hours ⎕ Less than 1hour	⎕ 8 horas ou mais ⎕ De 4 a 8 horas ⎕ De 1 a 4 horas ⎕ Menos de 1 hora
9	**Other than** music concerts and **headset use** (already covered in questions 3 and 8), how often did you listen to music, radio programs, etc. from audio speakers in a car or at home?	Além de shows de música e uso de headsets (já abordados nas questões 3 e 8), com que frequência você ouviu música, programas de rádio etc. em caixas de som no carro ou em casa?	**In addition to** music concerts and **the use of headsets** (already covered in questions 3 and 8), how often did you listen to music, radio programs, etc. from audio speakers in the car or at home?	Além de *shows* de música e uso de *headsets* (já abordados nas questões 3 e 8), com que frequência você ouviu música, programas de rádio etc. em caixas de som no carro ou em casa?
	⎕ Never ⎕ Every few months ⎕ Monthly ⎕ Weekly ⎕ Daily	⎕ Nunca ⎕ A cada 2 ou 3 meses ⎕ Mensalmente ⎕ Semanalmente ⎕ Diariamente	⎕ Never ⎕ Every few months ⎕ Monthly ⎕ Weekly ⎕ Daily	⎕ Nunca ⎕ A cada 2 ou 3 meses ⎕ Mensalmente ⎕ Semanalmente ⎕ Diariamente
	If you listened via speakers, on average, how many hours did each time/session last?	Se você ouviu em caixas de som, por quanto tempo, em média, você ouviu cada vez?	If you listened via speakers, on average, how many hours did you listen each time?	Se você ouviu em caixas de som, por quanto tempo, em média, você ouviu cada vez?
	⎕ 8 hours or more ⎕ 4 hours up to 8 hours ⎕ 1 hour up to 4 hours ⎕ Less than 1 hour	⎕ 8 horas ou mais ⎕ De 4 a 8 horas ⎕ De 1 a 4 horas ⎕ Menos de 1 hora	⎕ 8 hours or more ⎕ 4 hours up to 8 hours ⎕ 1 hour up to 4 hours ⎕ Less than 1 hour	⎕ 8 horas ou mais ⎕ De 4 a 8 horas ⎕ De 1 a 4 horas ⎕ Menos de 1 hora
10	**Now think back to this past summer**. Over the summer months, did you work **a noisy** paid **job**, such as in construction, farming, **a factory, lawn service, carwash, or other indoor or outdoor job working around loud** equipment or machinery? **By noisy job, we mean sounds so loud that you had** to shout or speak in a **raised voice to be heard at arm’s length.**	Ao longo do último ano, você trabalhou em algum serviço formal ou informal ruidoso, como em construção, fábrica, fazenda, jardinagem, lava-rápido, ou outro serviço em ambiente interno ou externo, próximo a equipamentos ou maquinário ruidosos? O que queremos dizer com serviço (ambiente) ruidoso é que o som seria tão intenso que você teria que gritar ou falar em um tom elevado para que lhe escutassem à distância de um metro.	**Over the past year,** did you work one **or more** noisy paid **jobs**, such as construction, farming, **factory, gardening, car wash, or other service indoors or outdoors close to noisy** equipment or machinery? What we mean by a noisy (ambient) service is that the sound would be so loud that you would have to shout or speak in a **high tone to be heard over a meter away.**	Ao longo do último ano, você trabalhou em algum serviço formal ou informal ruidoso, como em construção, fábrica, jardinagem, lava-rápido, ou outro serviço em ambiente interno ou externo, próximo a equipamentos ou maquinário ruidosos? O que queremos dizer com serviço (ambiente) ruidoso é que o som seria tão intenso que você teria que gritar ou falar em um tom elevado para que lhe escutassem à distância de um metro.
	⎕ Yes ⎕ No (if no, skip to # 11)	⎕ Sim ⎕ Não	⎕ Yes ⎕ No	⎕ Sim ⎕ Não
	If yes, please describe the noisy **job(s**): __________________________________	Se sim, por favor descreva o serviço (ambiente) ruidoso:_____________________________	If yes, please describe the noisy **service (environment)**:	Se sim, por favor descreva o serviço (ambiente) ruidoso:_____________________________
	If you worked **a noisy job**, please **estimate** the number of hours you worked in a typical week:	Se você trabalhou em um serviço (ambiente) ruidoso, por favor dê uma estimativa do número de horas que você trabalhou numa semana normal:	__________________________________	Se você trabalhou em um serviço (ambiente) ruidoso, por favor dê uma estimativa do número de horas que você trabalhou numa semana normal:
	________ **average hours** worked per **typical week during the school year**	________ horas trabalhadas por semana normal neste serviço.	If you worked in a noisy (environment) service, please give an estimate of the number of hours you worked in a typical week:	________ horas trabalhadas por semana normal neste serviço.
	If you worked **a noisy** job **during the school year, did your employer give you earplugs or earmuffs to wear** at work?	Se você trabalhou em um serviço ruidoso, o seu empregador lhe forneceu protetor auditivo (tampão de ouvido ou abafador) para usar no trabalho? ⎕ Sim ⎕ Não	________ **hours** worked per **week normal in this service**	Se você trabalhou em um serviço ruidoso, o seu empregador lhe forneceu protetor auditivo (tampão de ouvido ou abafador) para usar no trabalho? ⎕ Sim ⎕ Não
	⎕ Yes ⎕ No	Com que frequência você usou protetor auditivo (tampão de ouvido ou abafador) quando estava próximo a ruídos intensos nesse serviço?	If you worked **in a noisy** job, **have your employer provided you with earplugs or earmuffs to be used** at work?	Com que frequência você usou protetor auditivo (tampão de ouvido ou abafador) quando estava próximo a ruídos intensos nesse serviço?
	How often did you **wear earplugs or earmuffs when around loud noise at this noisy job**?	⎕ Nunca ⎕ Às vezes ⎕ Sempre	⎕ Yes ⎕ No	⎕ Nunca ⎕ Às vezes ⎕ Sempre
	⎕ Never ⎕ Sometimes ⎕ Always		How often did you **use earplugs or earmuffs when you were close to loud noises in this service**?	
			⎕ Never ⎕ Sometimes ⎕ Always	
11	Other than during the summer, over the past year, did you work one or more noisy paid jobs, such as in construction, farming, a factory, lawn service, carwash, or other indoor or outdoor job working around loud equipment or machinery? By noisy job, we mean sounds so loud that you had to shout or speak in a raised voice to be heard at arm’s length.	-	-	-
	⎕ Yes ⎕ No (if no, you’re done with the survey)			
	If yes, please describe the noisy job(s): __________________________________			
	If you worked a noisy job, please estimate the number of hours you worked in a typical week:			
	________ average hours worked per typical week during the school year			
	If you worked a noisy job during the school year, did your employer give you earplugs or earmuffs to wear at work? ⎕ Yes ⎕ No			
	How often did you wear earplugs or earmuffs when around loud noise at this noisy job?			
	⎕ Never ⎕ Sometimes ⎕ Always			

When translations were synthesized, it was decided to merge questions 10 and 11 into one (question 10), adjusting it to the Brazilian situation. These questions in the original NEQ use seasons of the year as a reference, addressing climatic conditions of the place of origin of the questionnaire and the local “summer job” tradition (Chart 1).

There was also a change in question 1 in NEQ-S, which dealt with firearm use - which in general is not part of the Brazilian culture. This question was completely changed, merging three other questions present in Appendix A of the reference article^(10)^ (Chart 2).

**Chart 2:** Questions in the 1-Minute Noise Screen or *Triagem de Exposição ao Ruído de 1-Minuto* in the translation and cross-cultural adaptation process

QUESTIONS - NEQ-S	ORIGINAL VERSION IN ENGLISH	SUMMARY OF TRANSLATIONS INTO BRAZILIAN PORTUGUESE	SUMMARY OF BACK TRANSLATIONS INTO ENGLISH	COMMITTEE OF EXPERTS: EQUIVALENCE
Instructions	**DURING THE PAST YEAR (12 months)**	**DURANTE O ÚLTIMO ANO (12 meses):**	**DURING THE PAST YEAR (12 months)**	**DURANTE O ÚLTIMO ANO (12 meses):**
1	How often **were you around or did you** shoot firearms such as rifles, pistols, shotguns, etc.?	Com que frequência você esteve **próximo a, ou disparou, armas de fogo tais como rifles, pistolas, espingarda etc.?**	How often **have you been close to, or** shoot firearms such as rifles, pistols, shotguns, etc.?	Com que frequência você esteve **exposto a sons intensos que fizeram você sentir zumbido, ouvidos tampados, dores ou incômodo nos ouvidos?**
	⎕ Never ⎕ Every few months ⎕ Monthly ⎕ Weekly ⎕ Daily	⎕ Nunca ⎕ A cada 2 ou 3 meses ⎕ Mensalmente ⎕ Semanalmente ⎕ Diariamente	⎕ Never ⎕ Every few months ⎕ Monthly ⎕ Weekly ⎕ Daily	⎕ Nunca ⎕ A cada 2 ou 3 meses ⎕ Mensalmente ⎕ Semanalmente ⎕ Diariamente
2	How often **were you exposed** to loud sounds while working on a **paid job**? **By loud sounds, we mean sounds so loud that you had to shout or speak in a raised voice to be heard at arm’s length.**	Com que frequência você esteve exposto a sons altos enquanto trabalhava em um emprego assalariado? O que queremos dizer com som alto é que você teria que gritar ou falar em um tom elevado para que lhe escutassem à distância de um metro.	How often **have you been** exposed to loud sounds while working on a **wage employment**? **What we mean by loud sound is that you would have to scream or speak in a high tone so that they can hear you at a distance of one meter**.	Com que frequência você esteve exposto a sons intensos enquanto trabalhava em um emprego assalariado? O que queremos dizer com som intenso é que você teria que gritar ou falar em um tom elevado para que lhe escutassem à distância de um metro.
	⎕ Never ⎕ Every few months ⎕ Monthly ⎕ Weekly ⎕ Daily	⎕ Nunca ⎕ A cada 2 ou 3 meses ⎕ Mensalmente ⎕ Semanalmente ⎕ Diariamente	⎕ Never ⎕ Every few months ⎕ Monthly ⎕ Weekly ⎕ Daily	⎕ Nunca ⎕ A cada 2 ou 3 meses ⎕ Mensalmente ⎕ Semanalmente ⎕ Diariamente
3	How ofte**n were you exposed to any other types of loud sounds, such as** power tools, **lawn equipment,** or loud music? **By loud sounds, we mean sounds so loud that you had to shout or speak in a raised voice to be heard at arm’s length.**	Com que frequência você esteve exposto a quaisquer outros tipos de som alto tais como ferramentas elétricas, máquinas de cortar grama, ou música alta? O que queremos dizer com som **alto** é que você teria que gritar ou falar em um tom elevado para que lhe escutassem à distância de um metro.	How often **have you been exposed to any other types of loud sounds such as** power tools, **lawn mowers,** or loud music? **What we want to say with a loud sound is that you would have to shout or scream in a high tone so that listened to you at a distance of one meter.**	Com que frequência você esteve exposto a quaisquer outros tipos de sons intensos tais como ferramentas elétricas, máquinas de cortar grama ou música alta? O que queremos dizer com som intenso é que você teria que gritar ou falar em um tom elevado para que lhe escutassem à distância de um metro.
	⎕ Never ⎕ Every few months ⎕ Monthly ⎕ Weekly ⎕ Daily	⎕ Nunca ⎕ A cada 2 ou 3 meses ⎕ Mensalmente ⎕ Semanalmente ⎕ Diariamente	⎕ Never ⎕ Every few months ⎕ Monthly ⎕ Weekly ⎕ Daily	⎕ Nunca ⎕ A cada 2 ou 3 meses ⎕ Mensalmente ⎕ Semanalmente ⎕ Diariamente

Moreover, the term “earplug” was adjusted to “hearing protection device”, according to our reality. Also, an explanatory glossary was added in parentheses: “earplugs or earmuffs” (Chart 1).

After the back-translation stage, the back-translated versions were compared with the original ones, highlighting differences such as verb tenses, conditional rules, false cognates, and synonyms. Then, in the expert committee analysis, these differences were reviewed, making the necessary adaptation of specific words (Charts 1 and 2).

In question 3 in NEQ, the word “religious” was included because such events use amplified sound and are commonly attended by Brazilians. In question 4, the words “buggy/kart” were included in the category of quadricycles because they are the most used names for them. In question 9, the term “sound speaker” was used instead of “music speaker” because they can be used for ends other than music, such as radio news and podcasts.

In the pretest, participants had no difficulties understanding and answering the questions in either questionnaire. This was true to both closed-ended and open-ended questions - i.e., 7 and 10, which asked them to describe the instrument they played (when applicable) or the services carried out in noisy environments.

After the pretest, the content and layout of the questionnaires were submitted for validation. In this stage, the reports with the comments of the target population and the observations of the researcher who accompanied the application of the instruments were presented to the expert committee. Some sentences and specific words needed small changes; they were adapted with experiential/semantic equivalence while maintaining them as close as possible to the original version. The final versions are presented in Appendices A and B.

Intra-subject NEQ reliability was tested by assessing two questions on noisy work (Question 10 in NEQ and Question 2 in NEQ-S). The kappa agreement test between these combined data was 0.550 (p < 0.001), indicating moderate agreement.

The internal consistency was analyzed with Cronbach's alpha coefficient, whose result was 0.711, indicating substantial internal consistency.

## DISCUSSION

Foreign instruments have been increasingly translated and cross-culturally adapted in the last years, enabling their use in other cultures. Hence, their data are ensured to express what they were meant to measure, making it possible to compare such data between different cultures that use standardized instruments. Moreover, they save the time and money spent on producing new instruments^(14,15)^.

There are currently various translation and cross-cultural adaptation strategies, in which all stages must be given due importance to minimize errors and losses regarding the original characteristics of the instruments - which may occur in such a process^(14,16)^. Although there is no gold-standard model of translation and cross-cultural adaptation, four essential stages (translation, back-translation, expert committee review, and pretest) are recommended to ensure the validity and reliability of the original instrument^(17)^.

According to the methodological reference^(11)^, which is widely used both nationally and internationally^(14)^, all the abovementioned stages were followed. They aimed at semantic, idiomatic, experiential, and conceptual equivalence between the original text and its translation, trying to solve the difficulties caused by multiple meanings and grammar issues that arose in the process and might have kept the target population from understanding the instrument.

Special care was taken when choosing translators and expert committee members, including professionals with expertise in Audiology and Occupational Health. The sample, in its turn, included different age groups and levels of educational attainment, either exposed or not to occupational noise, thus verifying whether the items were understandable - as well as the applicability of the instrument to a diversified sample, larger than commonly used in the literature^(11,15,16,18-21)^. Nevertheless, despite all the care taken in sample selection, there may have been some influence from selection bias, which is inherent to any research with a convenience sample.

After applying the pretest, some changes were made to NEQ and NEQ-S questions regarding punctuation, context, and the literal translation process. Two important changes were necessary, namely: merging questions 10 and 11 into one in NEQ (question 10) and replacing items in question 1 in NEQ-S with some of those in the appendix of the original instrument. These changes were made to ensure the compatibility of the questions with the Brazilian reality, making them easier for the target population to understand. It must be pointed out that the expert committee proposed merging questions 10 and 11 of the original instrument to adapt them to the Brazilian reality (climate and tradition), as summer jobs are not usual in our culture. Likewise, question 1 in NEQ-S had to be changed because this screening is meant to indicate the risk for NIHL based on the score of three questions. Since hunting and using firearms are not usual for most Brazilians, the expert committee decided to use another three questions taken from an additional instrument developed by the authors^(10)^, which investigate symptoms (tinnitus, “full” ear feeling, earaches, or discomfort) that may be present after exposure to intense noise.

The need to change questions or statements from original instruments is reported by researchers in the area, who emphasize that cultural differences may require such changes, especially when they involve specific conditions, as previously mentioned^(22)^ and observed in the present study.

NEQ and NEQ-S are relatively new instruments, published in 2017^(10)^. Therefore, no other studies on their translation and cross-cultural adaptation to other languages and countries were found, preventing comparison with other versions.

Regarding the intra-subject reliability of the instrument, the original study^(10)^ used the same methodology, comparing two repeated questions on the same topic (noisy work). Their results were similar to ours (kappa = 0.590 - moderate agreement).

Cronbach’s alpha coefficient was 0.711, indicating substantial internal consistency in this instrument analysis. This is a reliability measure that reflects how questionnaire items are mutually related. It is important and desirable that this value be between 0.70 and 0.95^(23)^, as the one verified in this study.

Even though other instruments quantify yearly noise exposure^(24)^, NEQ uses simple task-based questions. Hence, it can be used to estimate people’s yearly exposure to either occupational or non-occupational noise. It also has the screening version (NEQ-S), which quickly and easily identifies individuals at risk of NIHL.

Some studies have been using NEQ to characterize participants’ doses of noise exposure - e.g., Grinn et al.^(25)^, Spankovich et al.^(26)^, Bernard et al.^(27)^, Athirah and Shahida^(28)^, Powell^(29)^. They used this instrument to calculate cumulated yearly doses of noise based on self-reported activities.

Hence, standardizing new instruments and making them available are important strategies to develop science and have them used by health professionals, with an impact on clinical practice. They may also be useful as screening instruments to identify harmful day-to-day noises, helping better plan interventions, especially in the occupational area^(10,16,19,20)^.

In the next stage of our study, the translated and adapted instrument will be applied in order to determine NEQ diagnostic values (sensitivity, specificity, and accuracy), comparing it with the gold-standard examination (audiometry) to identify NIHL.

## CONCLUSION

The translation and cross-cultural adaptation were made according to the methodology indicated in the national and international literature. They followed the stages of translation, back-translation, expert committee, and pretest, including the equivalences necessary to maintain the face and content validity of the original instrument. Making NEQ and NEQ-S available in Brazilian Portuguese opens new fields of research to address yearly noise exposure quantification more in-depth.

## Appendix A Final version: Noise Exposure Questionnaire - NEQ (Johnson et al. ^(10)^) translated and adapted to Brazilian Portuguese.)

Noise Exposure Questionnaire - NEQ

**Table:**

Please answer the following questions about yourself, your hearing, and any noise you may have been around during the past year. Write an answer in the blank [______] or check [ x ] the best answer to each question.	
Please answer these questions about any loud sounds. DURING THE PAST YEAR (12 months):	
1	Other than in your paid work (considering housework), how often have you used electrical tools, chainsaws, or other noisy working tools?
	□ Never □ Every 2 or 3 months □ Monthly □ Weekly □ Daily
	If you used electrical tools, for how long, on average, did you use them each time?
	□ For 8 or more hours □ For 4 to 8 hours □ For 1 to 4 horas □ Less than 1 hour
	If you used electrical tools, how often did you use hearing protection devices (earplugs or earmuffs) during the activity?
	□ Never □ Sometimes □ Always
2	Other than in your paid work (considering housework), how often have you driven heavy vehicles or operated noisy machines (e.g., tractors, trucks, farm or yard machines quintal, such as lawn mowers, leaf blowers, and blower vacs)?
	□ Never □ Every 2 or 3 months □ Monthly □ Weekly □ Daily
	If you drove/operated noisy machines, for how long, on average, did you do it each time?
	□ 8 or more hours □ For 4 to 8 hours □ For 1 to 4 hours □ Less than 1 hour
	If you drove/operated noisy machines, how often did you use hearing protection devices (earplugs or earmuffs) during the activity?
	□ Never □ Sometimes □ Always
3	How often have you gone to car or truck races, sports, commercial, school, or religious events, music concerts or shows, parties, or any other events with loudspeakers used to make announcements or play music?
	□ Never □ Every 2 or 3 months □ Monthly □ Weekly □ Daily
	If you went to such events, how long did each one last on average?
	□ 8 or more hours □ For 4 to 8 hours □ For 1 to 4 hours □ Less than 1 hour
	If you went to such events, how often did you use hearing protection devices (earplugs or earmuffs) during the activity?
	□ Never □ Sometimes □ Always
4	***Please, continue answering the following questions regarding any intense sound.***
	**OVER THE LAST YEAR (12 months):**
	Other than cars, how often did you drive or operated motor vehicles, such as motorcycles, jet-skis, speedboats, all-terrain vehicles, buggies, or karts?
	□ Never □ Every 2 or 3 months □ Monthly □ Weekly □ Daily
	If you drove motor vehicles, for how long, on average, did you drive them each time?
	□ 8 or more hours □ For 4 to 8 hours □ For 1 to 4 hours □ Less than 1 hour
	If you drove motor vehicles, how often did you use hearing protection devices (earplugs or earmuffs) during the activity?
	□ Never □ Sometimes □ Always
5	How often did you fly in or pilot a small or private airplane?
	□ Never □ Every 2 or 3 months □ Monthly □ Weekly □ Daily
	If you flew in one of these airplanes, how long did each flight last on average?
	□ 8 or more hours □ For 4 to 8 hours □ For 1 to 4 hours □ Less than 1 hour
	If you flew in one of these airplanes, how often did you use hearing protection devices (earplugs or earmuffs) during the activity?
	□ Never □ Sometimes □ Always
6	How often did you fire or were near firearms, such as rifles, pistols, shotguns, etc.?
	□ Never □ Every 2 or 3 months □ Monthly □ Weekly □ Daily
	If you fired or were near firearms, how many shots, on average, did you give or witness each time?
	_________ shotguns/rifle shots each time. _________ pistol shots each time.
	If you fired or were near firearms, how often did you use hearing protection devices (earplugs or earmuffs) during the activity?
	□ Never □ Sometimes □ Always
7	How often did you play a musical instrument?
	□ Never □ Every 2 or 3 months □ Monthly □ Weekly □ Daily
	If you played an instrument, please, tell us which one it was: __________________________________
	If you played a musical instrument, for how long did you play it on average?
	□ 8 or more hours □ For 4 to 8 hours □ For 1 to 4 hours □ Less than 1 hour
	If you played a musical instrument, how often did you use hearing protection devices (earplugs or earmuffs) while you play it?
	□ Never □ Sometimes □ Always
	***Please, continue answering the following questions regarding any intense sound.***
	**OVER THE LAST YEAR (12 months):**
8	How often did you listen to music, radio programs, etc., wearing earphones?
	□ Never □ Every 2 or 3 months □ Monthly □ Weekly □ Daily
	If you wore earphones, for how long, on average, did you use them each time?
	□ 8 or more hours □ For 4 to 8 hours □ For 1 to 4 hours □ Less than 1 hour
9	Apart from music concerts and wearing earphones (already approached in questions 3 and 8), how often did you listen to music, radio programs, etc. on loudspeakers in the car or at home?
	□ Never □ Every 2 or 3 months □ Monthly □ Weekly □ Daily
	For how long, on average, did you listen to it each time?
	□ 8 or more hours □ For 4 to 8 hours □ For 1 to 4 hours □ Less than 1 hour
10	Over the last year, did you work in any noisy formal or informal job, such as construction, factory, gardening, carwash, or other indoor or outdoor work near noisy equipment or machines? By intense sound in the noisy work setting, we mean it was so intense that you would need to shout or speak loudly for someone to hear you one meter away.
	□ Yes □ Νο
	If so, please describe the noisy work (setting):
	____________________________________________________________________________
	If you worked in a noisy work (setting), please estimate the number of hours you worked in a normal week:
	________ hours worked in a normal week in this job.
	If you worked in a noisy job (setting), did your employer provide you with hearing protection (earplugs or earmuffs) to wear at work? ð Yes ð No
	How often did you wear hearing protection (earplugs or earmuffs) when you were near intense noises at work?
	□ Never □ Sometimes □ Always

## Appendix B Final version: 1-Minute Noise Screen (Johnson et al.^(10)^ ) translated and adapted to Brazilian Portuguese.)

**Table:** 1-Minute Noise Screening

OVER THE LAST YEAR (12 months):			
1	How often were you exposed to intense sounds that made you have tinnitus, clogged ears, or pain or discomfort in your ears? By intense sound, we mean you would have to shout or speak loudly for someone to hear you one meter away.		
	□ Never □ Every 2 or 3 months □ Monthly □ Weekly □ Daily		
2	How often were you exposed to intense sounds while working in paid work? By intense sound, we mean you would have to shout or speak loudly for someone to hear you one meter away.		
	□ Never □ Every 2 or 3 months □ Monthly □ Weekly □ Daily		
3	**Other than in your paid work**, how often were you exposed to any other type of intense sound, such as electric tools, loud music, or lawn mowers? By intense sound, we mean you would have to shout or speak loudly for someone to hear you one meter away.		
	□ Never □ Every 2 or 3 months □ Monthly □ Weekly □ Daily		
			**Noise exposure score:**
			**__________**

How to Calculate Your

1-Minute Noise Screening Score

First, ascribe the scores below to your answers to each question:

**Table:**

	Never	Every 2 or 3 months	Monthly	Weekly	Daily
Question 1.	0	1	2	3	4
Question 2.	0	1	2	3	4
Question 3.	0	1	2	3	4

Then, sum the three individual scores to obtain the total noise exposure score. Write down the total score in the box in the lower right corner of your card.

Read, in the back of this sheet, the explanation of your noise exposure score, as well as suggestions on how to manage the risk of developing noise-induced hearing loss.

Example:

1-Minute Noise Screening: Recommendations

**Table:**

If your noise score is in this range:	Your risk is:	Explanation
0 to 4	Lower risk	Based on your experience with noise over the last year, your risk of developing noise-induced hearing loss is relatively low - as long as you continue to interact with such noise levels in the future. However, if your exposure increases, your risk of developing hearing loss will also increase.
		Each person’s tolerance to noise is different, and their susceptibility can hardly be foreseen. Even so, it is important to remember that the risk increases as the sound gets more intense and you spend more time exposed to it and more often. See the hints below to learn how to manage the risk of developing noise-induced hearing loss.

		**Special observation to those who used firearms**: If you use firearms, you are at a high risk of hearing loss, even if you use them every 2 or 3 months and your score is low in the 1-Minute Noise Screening. See the hints below to learn what you can do to manage this risk.
5 or more	Higher risk	Based on your experience with noise over the last year, you are at risk of developing noise-induced hearing loss if you continue to interact with such or higher noise levels in the future.
		Each person’s tolerance to noise is different, and their susceptibility can hardly be foreseen. Even so, it is important to remember that the risk increases as the sound gets more intense and you spend more time exposed to it and more often. See the hints below to learn how to manage the risk of developing noise-induced hearing loss.

What You Can Do to Manage the Risk:

**Avoid intense noises whenever possible:** It may seem redundant to say it, but avoiding intense noises is the first step to preserving your hearing for your whole life. Remember, if you need to shout for someone to hear you one or two meters away, the background noise is probably at a dangerous level. Look for quieter products when shopping for noisy tools or equipment, such as leaf blowers, blower vacs, or lawn mowers. Also, turn down the volume when you are using electronic devices, such as mobiles and music players.

**Wear hearing protection whenever you are near loud noises**: When you cannot avoid intense noises, wear well-fitted hearing protection (earplugs or earmuffs), even in occasional circumstances. Hearing protection can be purchased in various drug, convenience, hardware, and sports stores. Make sure you are well-trained to wear and care for your hearing protection devices and replace them when necessary. The adequate and consistent use of hearing protection can minimize your risk, especially if you use firearms. In this case, exposure to a single shot may be enough to damage your hearing if you are not wearing hearing protection.

**Have your hearing regularly tested:** Keep an eye on your hearing! Have it tested routinely - once a year if you are in the higher-risk group listed above or if you increase your exposure to noise. Follow up on your hearing test results and ask your audiologist to compare your annual tests with the most recent ones to verify any significant changes that may pose a concern.

**Take care of your ears:** Go see a doctor if you notice problems such as sudden hearing changes, pain, ear fullness, or tinnitus.
