# Supplementary material for: Translation and cross-cultural adaptation of the Noise Exposure Questionnaire (NEQ) to Brazilian Portuguese
Source: Codas. 2023 Jul 7;35(3):e20220062. doi: 10.1590/2317-1782/20212022062en (PMC10348664; doi:10.1590/2317-1782/20212022062en)
Supplement: Translation

# Translation: Tradução e adaptação transcultural do *Noise Exposure Questionnaire* (NEQ) para o português brasileiro

**DOI:** 10.1590/2317-1782/20212022062pt

**Authors:** Letícia Campos de Oliveira, Clayton Henrique Rocha, Carla Gentile Matas, Karina Mary de Paiva, Renata Rodrigues Moreira, Alessandra Giannella Samelli

**Affiliations:** 1 Departamento de Fisioterapia, Fonoaudiologia e Terapia Ocupacional, Faculdade de Medicina - FMUSP, Universidade de São Paulo - São Paulo (SP), Brasil.; 2 Departamento de Fonoaudiologia, Universidade Federal de Santa Catarina - Florianópolis (SC), Brasil.; 3 Hospital Universitário - HU, Universidade de São Paulo - São Paulo (SP), Brasil.

**Keywords:** Tradução, Audição, Perda Auditiva Provocada por Ruído, Ruído, Questionários

## Abstract

**Objetivo:**

Traduzir e realizar a adaptação transcultural dos instrumentos Noise Exposure Questionnaire (NEQ) e 1-Minute Noise Screen (NEQ-S) para a versão em português brasileiro.

**Método:**

O processo de tradução e adaptação transcultural seguiu procedimentos amplamente utilizados em pesquisas da área da saúde, compostos pelas seguintes etapas: tradução inicial, síntese das traduções, retrotradução, comitê de especialistas, pré-teste, e validação do conteúdo e aparência. Na etapa de pré-teste, participaram 60 trabalhadores, que responderam aos questionários e, posteriormente, os avaliaram quanto ao: entendimento, aparência, clareza e escrita. Para a verificação da confiabilidade, foi utilizado o teste kappa de Cohen e para a análise da consistência interna foi aplicado o coeficiente alfa de Cronbach.

**Resultados:**

As versões traduzidas e adaptadas dos questionários NEQ e NEQ-S mostraram-se semelhantes em relação ao significado geral e referencial. Entretanto, algumas modificações e adaptações foram realizadas, para adequação à realidade brasileira. O teste kappa indicou concordância moderada e o coeficiente alfa de Cronbach, consistência interna substancial.

**Conclusão:**

A tradução e a adaptação transcultural foram realizadas de acordo com a metodologia preconizada na literatura nacional e internacional, incluindo-se as equivalências necessárias para a manutenção da validade de face e conteúdo com o instrumento original. A disponibilização dos questionários NEQ e NEQ-S na versão da língua portuguesa brasileira abre novos campos de pesquisa para aprofundamento da quantificação de exposição anual ao ruído.

## INTRODUÇÃO

Estudos voltados ao conhecimento quanto aos danos auditivos relacionados ao ruído têm aumentado a cada dia e acredita-se que este cenário seja decorrente dos importantes resultados de pesquisas envolvendo cobaias^(1)^, que demonstraram sinaptopatia coclear induzida por ruído^(2)^. Nestes estudos com cobaias, as variáveis de exposição ao ruído podem ser cuidadosamente controladas (frequência, nível de intensidade e duração da exposição), permitindo estimativas precisas da associação entre exposição ao ruído e danos auditivos^(3)^. Em estudos com humanos, a técnica recomendada em avaliações prospectivas destas variáveis é a dosimetria; contudo, as estimativas retrospectivas dependem predominantemente do autorrelato da exposição cumulativa ao ruído, sendo o questionário o instrumento indicado para obter estas informações^(2,3)^.

Estima-se que, nos Estados Unidos, aproximadamente 27,7 milhões de indivíduos com idades entre 20 e 69 anos vivam com perda auditiva induzida por ruído (PAIR)^(4)^. Além disso, a PAIR continua sendo a segunda doença ocupacional autorreferida mais comum, apesar da existência de regulamentações e intervenções nos locais de trabalho^(5,6)^.

A alta prevalência da PAIR tem sido associada ao aumento da industrialização, juntamente com as dificuldades na formulação e implementação de políticas públicas e de medidas preventivas adequadas contra o ruído, além das dificuldades relacionadas aos sistemas de informação e coleta de dados para geração de indicadores consistentes e comparáveis^(7)^.

Sendo assim, em virtude das evidências ainda limitadas relacionadas à prevenção e controle da PAIR, bem como em virtude dos índices elevados de PAIR no mundo, é necessário o desenvolvimento de mais estudos na área, incluindo o desenvolvimento de instrumentos para estimar a exposição ao ruído ocupacional e não ocupacional, uma vez que ainda existem lacunas. Dentre elas, a falta de padronização nos procedimentos para coleta de informações autorreferidas; nas estimativas de risco auditivo (definição de fatores de risco não-ocupacionais); e na definição de instrumentos validados (acesso ao instrumento ou às instruções para o uso)^(2)^.

A padronização dos instrumentos, dentro do processo do autorrelato, pode minimizar os efeitos subjetivos da percepção do risco, já que se observa concepções diferentes dos riscos a que estão expostos em trabalhadores desempenhando funções idênticas em locais comuns. Suas percepções se fundamentam nos seus conhecimentos práticos, deduções e conversas com colegas de trabalho, bem como de informações recebidas da empresa^(2,8,9)^.

Nesta perspectiva, o “*Noise Exposure Questionnaire (NEQ)*” foi desenvolvido como um questionário detalhado baseado em tarefas, buscando quantificar a história de exposição ao ruído de um indivíduo, proveniente de fontes ocupacionais e não ocupacionais. Além disso, foi desenvolvido um screening “*1-Minute Noise Screen (NEQ-S*), para a identificação de indivíduos com maior risco de desenvolverem PAIR^(10)^.

Desta forma, em virtude da escassez de ferramentas deste tipo no Brasil, o objetivo da presente pesquisa foi traduzir e realizar a adaptação transcultural das ferramentas *NEQ e NEQ-S* para o português brasileiro.

## MÉTODO

Este trabalho foi aprovado pelo Comitê de Ética da instituição (nº 858/08). Além disso, o uso do instrumento *Noise Exposure Questionnaire* (NEQ) para este fim foi autorizado por uma das autoras.

### Descrição do instrumento

O NEQ original é composto por 10 questões que visa estimar a exposição anual ao ruído de um indivíduo. Por isso, as alternativas de resposta variam entre “Nunca; A cada 2 ou 3 meses; Mensalmente; Semanalmente; Diariamente”; “8 horas ou mais; De 4 a 8 horas; De 1 a 4 horas; Menos de 1 hora”; referentes a diversas situações de exposição ao ruído, bem como “Nunca; Às vezes; Sempre”, quando diz respeito ao uso de dispositivos de proteção individual. Além disso, existe um instrumento de *screening* (o NEQ-S ou 1-*Minute Noise Screen*), que pode ser utilizado para estimar o risco do indivíduo em desenvolver perda auditiva induzida por ruído (PAIR). Este *screening* é composto por três questões com as seguintes alternativas: “Nunca; A cada 2 ou 3 meses; Mensalmente; Semanalmente; Diariamente”; que são pontuadas de 0 a 4, respectivamente. Pontuações no *screening* iguais ou maiores que 5 indicam risco mais elevado para o desenvolvimento de PAIR.

### Procedimentos

Os participantes que aceitaram participar da pesquisa, assinaram o Termo de Consentimento Livre e Esclarecido.

O estudo foi realizado entre março de 2020 e dezembro de 2020. O processo de tradução e adaptação transcultural contou com procedimentos amplamente utilizados na literatura, para a área da saúde^(11,12)^.

A tradução e adaptação transcultural do instrumento foram realizadas em etapas: tradução inicial, síntese das traduções, retrotradução, comitê de especialistas, pré-teste, e, validação do conteúdo e aparência. A seguir foram descritas as etapas^(11,12)^ (Figura 1):

**Figura 1:** Fluxograma - etapas da tradução e adaptação transcultural do instrumento NEQ

- Tradução e síntese das traduções: foram realizadas duas traduções independentes por dois tradutores diferentes, fluentes no idioma inglês: um deles era fonoaudiólogo, com experiência em tradução, e o outro não possuía conhecimento específico na área, gerando as versões T1 e T2. Em seguida, realizou-se a síntese das traduções por meio de uma reunião de consenso entre os dois tradutores e quatro pesquisadores fonoaudiólogos que atuam na área. Nesse momento, foram realizadas as reformulações necessárias, originando a versão síntese das traduções iniciais, sendo chamada de T12.
- Retrotradução: foi realizada a retrotradução da versão T12, do idioma português para o inglês, por outros dois tradutores independentes bilíngues que não tinham conhecimento do instrumento original. Cada retrotradutor produziu uma nova versão, chamadas de BT1 e BT2. Esta etapa teve o objetivo de avaliar se o conteúdo da versão síntese era semelhante ao do instrumento original.
- Comitê de especialistas: esta reunião teve o objetivo de produzir a versão final preliminar do instrumento, a ser usada no “pré-teste”. Foi realizada a análise das equivalências semântica (vocabulário e gramática através dos dois idiomas, analisando o significado referencial - denotativo - e o significado geral - conotativo), idiomática (propondo substituições para expressões idiomáticas difíceis de serem traduzidas), experiencial (propondo substituições para experiências não disponíveis no país) e linguística ou conceitual (para palavras com diferentes significados nas diferentes culturas) das versões traduzidas, em comparação aos questionários originais em inglês.
- Pré-teste: As versões finais preliminares foram utilizadas em um pré-teste para testar a clareza das perguntas e a facilidade de respostas, em um grupo de trabalhadores, com a finalidade de identificar possíveis problemas na compreensão dos questionários.
  • Etapa final: Após a aplicação do pré-teste, foram submetidos todos os relatórios realizados ao longo de todo processo ao comitê de especialistas, acompanhado da versão traduzida preliminar, com o objetivo de verificar se as etapas recomendadas foram seguidas, e se os relatórios refletem o processo. Conforme a necessidade, a partir dos resultados do pré-teste, pequenos ajustes finais aos questionários foram propostos.

Para garantir a adequação da etapa de pré-teste, procedeu-se à aplicação do instrumento numa amostra de indivíduos com características semelhantes àqueles a quem se destina o instrumento^(11)^. Os critérios de inclusão da casuística foram: indivíduos com idade acima de 18 anos; de ambos os sexos; ser trabalhador ativo da universidade onde o estudo foi desenvolvido; saber ler e escrever; não apresentar distúrbios neurológicos, cognitivos e/ou psiquiátricos que pudessem interferir na compreensão das questões; ter realizado audiometria no serviço de audiologia da instituição com intervalo inferior a um ano. Os critérios de exclusão foram: possuir qualquer fator limitante que impossibilitasse a leitura e o preenchimento dos questionários; apresentar laudo audiométrico de perda auditiva do tipo condutiva.

Participaram desta etapa 62 trabalhadores de áreas diversas (administração, cozinha, manutenção, serralheria, marcenaria, serviços gerais, construção civil e engenharia) da instituição onde o estudo foi desenvolvido (universidade pública), conforme recomendado pelo referencial metodológico utilizado^(11)^. Houve a exclusão de dois participantes por não apresentarem o exame audiológico no período exigido. Desta forma, a amostra final contou com um total de 60 participantes.

Os trabalhadores foram contactados e convidados a participar da pesquisa. Após o aceite e assinatura do TCLE, os participantes responderam aos questionários completos (incluindo o NEQ e o NEQ-S). Posteriormente, os indivíduos avaliaram os questionários quanto ao: entendimento, aparência, clareza e escrita. Também foram incentivados a fornecer sugestões de melhorias, quando considerassem pertinentes.

### Análise estatística

Para a verificação da confiabilidade, foi utilizado o teste kappa de Cohen e para a análise da consistência interna foi aplicado o coeficiente alfa de Cronbach. Valores de alfa (ou kappa) menores do que 0,21 indicam consistência interna (ou concordância) fraca; de 0,21 a 0,40 razoável; de 0,41 a 0,60 moderada; de 0,61 a 0,80 substancial e maior do que 0,80, quase perfeita^(13)^.

## RESULTADOS

### Caracterização da casuística

Os 60 participantes tinham média de idade de 44 anos (DP=12; Mín: 22; Máx: 65), sendo 70% do sexo masculino. Com relação à escolaridade, a maioria (47%) possuía ensino superior completo, seguido por 43% com ensino médio completo; e 10% dos participantes com ensino fundamental completo.

Em relação as características principais da exposição ao ruído, observou-se que 52% têm exposição a ruído ocupacional e 48% têm exposição a ruído não ocupacional. Quanto ao laudo audiométrico, 68% dos trabalhadores avaliados apresentaram limiares auditivos dentro do padrão de normalidade (até 25dBNA) para a orelha esquerda e 63% para a orelha direita (Tabela 1).

**Tabela 1:** Caracterização da amostra quanto a exposição ao ruído, uso de DPA e laudo audiométrico

**Variável**	**Exposição**	**N**	**Percentual**
Ruído Ocupacional	Não	29	48%
	Sim	31	52%
**DPA**	Não	43	72%
	Sim	17	28%
**Ruído não ocupacional**	Não	19	32%
	Sim	41	48%
**Audiometria O.D**			
Audição normal*		38	63%
Perda auditiva		22	37%
**Audiometria O.E**			
Audição normal*		41	68%
Perda auditiva		19	32%

* Limiares auditivos menores ou iguais a 25 dB(NA), em todas as frequências avaliadas em conformidade com a Portaria nº 19^(30)^)

**Legenda:** DPA = Uso do dispositivo de proteção auditiva; O.D = Orelha direita; O.E = Orelha esquerda

### Questionários

Em geral, as versões traduzidas e retrotraduzidas do *NEQ* e *NEQ-S* mostraram-se semelhantes em relação ao significado geral e referencial. Entretanto, algumas modificações (adaptações, exclusões e inserções de palavras e frases) foram realizadas, uma vez que algumas questões não retratavam a realidade brasileira, e as divergências foram sanadas por consenso pelo comitê de especialistas, com o intuito de facilitar a compreensão dos questionários pela população estudada.

As mudanças realizadas relacionaram-se à presença de situações do idioma nativo, coloquialismos, expressões verbais e a possibilidade de mais de uma tradução.

As versões originais, síntese das traduções, síntese das retrotraduções e versão preliminar (após análise do comitê de especialistas) foram apresentadas nos Quadros 1 e 2.

**Quadro 1:** Perguntas do questionário NEQ - *Noise Exposure Questionnaire* ou Questionário de Exposição ao Ruído durante o processo de tradução e adaptação transcultural

QUESTÕES - NEQ	VERSÃO ORIGINAL EM INGLÊS	SÍNTESE DAS TRADUÇÕES PARA O PORTUGUÊS BRASILEIRO	SÍNTESE DAS RETROTRADUÇÕES PARA O INGLÊS	COMITÊ DE ESPECIALISTAS: EQUIVALÊNCIA SEMÂNTICA, IDIOMÁTICA, EXPERENCIAL E LINGUISTICA - VERSÃO PRELIMINAR
INSTRUÇÕES	Please answer the following questions about yourself, **your hearing, and any noise you may have been around during** the past year. Write **an** answer **in the blank** [______] or **check** [ x ] the best answer **to** each question.	Por favor, responda as questões a seguir sobre si mesmo, sobre sua audição, e sobre qualquer ruído a que tenha sido exposto ao longo do último ano. Escreva sua resposta na lacuna [______] ou assinale [x ] a melhor resposta em cada questão.	Please answer the following questions about yourself, **about your hearing, and about any noise that you have been exposed over** the past year. Write **your** answer **on** [______] or **tick** [ ] the best answer **for** each question.	Por favor, responda as questões a seguir sobre si mesmo, sobre sua audição, e sobre qualquer ruído a que tenha sido exposto ao longo do último ano. Escreva sua resposta na lacuna [______] ou assinale [ x ] a melhor resposta em cada questão.
	Please answer these questions about any loud sounds. DURING THE PAST YEAR (12 months):	Por favor, responda estas perguntas referentes a qualquer som intenso.	Please answer these questions about any loud sounds. DURING THE PAST YEAR (12 months):	Por favor, responda estas perguntas referentes a qualquer som intenso.
		DURANTE O ÚLTIMO ANO (12 meses):		DURANTE O ÚLTIMO ANO (12 meses):
1	Outside of a paid job, how often did you use power tools, **chainsaws**, or other shop tools?	Sem ser em trabalho assalariado, com que frequência você usou ferramentas elétricas, serras elétricas, ou outras ferramentas de oficina?	Outside of a paid job, how often did you use power tools, **saws electrical**, or other tools?	Sem ser em trabalho assalariado, com que frequência você usou ferramentas elétricas, serras elétricas, ou outras ferramentas de oficina?
	⎕ Never ⎕ Every few months ⎕ Monthly ⎕ Weekly ⎕ Daily	⎕ Nunca ⎕ A cada 2 ou 3 meses ⎕ Mensalmente ⎕ Semanalmente ⎕ Diariamente	⎕ Never ⎕ Every few months ⎕ Monthly ⎕ Weekly ⎕ Daily	⎕ Nunca ⎕ A cada 2 ou 3 meses ⎕ Mensalmente ⎕ Semanalmente ⎕ Diariamente
	If you used power tools, on average, how many hours did each time/session last?	Se você usou ferramentas elétricas, por quanto tempo, em média, você as usou cada vez?	If you used power tools, on average, how many hours did each time/session last?	Se você usou ferramentas elétricas, por quanto tempo, em média, você as usou cada vez?
	⎕ 8 hours or more ⎕ 4 hours up to 8 hours ⎕ 1 hour up to 4 hours ⎕ Less than 1 hour	⎕ 8 horas ou mais ⎕ De 4 a 8 horas ⎕ De 1 a 4 horas ⎕ Menos de 1 hora	⎕ 8 hours or more ⎕ 4 hours up to 8 hours ⎕ 1 hour up to 4 hours ⎕ Less than 1 hour	⎕ 8 horas ou mais ⎕ De 4 a 8 horas ⎕ De 1 a 4 horas ⎕ Menos de 1 hora
	If you used power tools, how often did you wear earplugs or earmuffs during this activity?	Se você usou ferramentas elétricas, com que frequência você usou protetor auditivo **(tampão de ouvido ou abafador)** durante a atividade?	If you used power tools, how often did you wear earplugs or earmuffs during this activity?	Se você usou ferramentas elétricas, com que frequência você usou protetor auditivo **(tampão de ouvido ou abafador)** durante a atividade?
	⎕ Never ⎕ Sometimes ⎕ Always	⎕ Nunca ⎕ Às vezes ⎕ Sempre	⎕ Never ⎕ Sometimes ⎕ Always	⎕ Nunca ⎕ Às vezes ⎕ Sempre
2	Outside of a paid job, how often did you drive heavy **equipment or use loud** machinery (such as tractors, trucks, **or farming or lawn equipment like mowers/leaf blowers)**?	Sem ser em trabalho assalariado, com que frequência você dirigiu veículos pesados ou operou maquinários ruidosos (tais como trator, caminhão, maquinário de fazenda ou de quintal como cortador de grama ou soprador / aspirador de folhas)?	Outside of a paid job, how often did you drive heavy **vehicles or operated noisy** machinery (such as tractor, truck, **farm or yard machinery such as lawn mower or leaf blower/ vacuum cleaner)**?	Sem ser em trabalho assalariado, com que frequência você dirigiu veículos pesados ou operou maquinários ruidosos (tais como trator, caminhão, maquinário de fazenda ou de quintal como cortador de grama ou soprador / aspirador de folhas)?
	⎕ Never ⎕ Every few months ⎕ Monthly ⎕ Weekly ⎕ Daily	⎕ Nunca ⎕ A cada 2 ou 3 meses ⎕ Mensalmente ⎕ Semanalmente ⎕ Diariamente	⎕ Never ⎕ Every few months ⎕ Monthly ⎕ Weekly ⎕ Daily	⎕ Nunca ⎕ A cada 2 ou 3 meses ⎕ Mensalmente ⎕ Semanalmente ⎕ Diariamente
	If you **drove/used loud machinery, on average, how many hours did each time/session last**?	Se você dirigiu/operou maquinário ruidoso, por quanto tempo, em média, você o usou cada vez?	If you **have drove/operated noisy machinery, how long, on average, have you used it each time**?	Se você dirigiu/operou maquinário ruidoso, por quanto tempo, em média, você o usou cada vez?
	⎕ 8 hours or more ⎕ 4 hours up to 8 hours ⎕ 1 hour up to 4 hours ⎕ Less than 1 hour	⎕ 8 horas ou mais ⎕ De 4 a 8 horas ⎕ De 1 a 4 horas ⎕ Menos de 1 hora	⎕ 8 hours or more ⎕ 4 to 8 hours ⎕ 1 to 4 hours ⎕ Less than 1 hour	⎕ 8 horas ou mais ⎕ De 4 a 8 horas ⎕ De 1 a 4 horas ⎕ Menos de 1 hora
		Se você dirigiu/operou maquinário ruidoso, com que frequência você usou protetor auditivo (tampão de ouvido ou abafador) durante a atividade?		Se você dirigiu/operou maquinário ruidoso, com que frequência você usou protetor auditivo (tampão de ouvido ou abafador) durante a atividade?
	If you **drove/used machinery, how often did you wear earplugs or earmuffs during this activity**?	⎕ Nunca ⎕ Às vezes ⎕ Sempre	If you **drove/operated noisy machinery, how often did you use hearing protection equipment (earplugs or earmuffs) during the activity**?	⎕ Nunca ⎕ Às vezes ⎕ Sempre
	⎕ Never ⎕ Sometimes ⎕ Always		⎕ Never ⎕ Sometimes ⎕ Always	
3	How often did you attend car/truck races, commercial/**high schoo**l sporting events, music **concerts/dances** or any other events with amplified **public announcement (PA)/music systems**?	Com que frequência você foi a corridas de carros/caminhões, eventos esportivos comerciais/escolares, concertos/*shows* de música/festas ou quaisquer outros eventos com sistema de som amplificado usado para fazer anúncios ou tocar músicas?	How often did you attend **to** car/truck races, commercial/**school/religious** sporting events, concerts/**parties** or any other events with an amplified **sound system used to advertise or to play music**?	Com que frequência você foi a corridas de carro/caminhões, eventos esportivos comerciais/escolares/**religiosos**, concertos/*shows* de música/festas ou quaisquer outros eventos com sistema de som amplificado usado para fazer anúncios ou tocar músicas?
	⎕ Never ⎕ Every few months ⎕ Monthly ⎕ Weekly ⎕ Daily	⎕ Nunca ⎕ A cada 2 ou 3 meses ⎕ Mensalmente ⎕ Semanalmente ⎕ Diariamente	⎕ Never ⎕ Every few months ⎕ Monthly ⎕ Weekly ⎕ Daily	⎕ Nunca ⎕ A cada 2 ou 3 meses ⎕ Mensalmente ⎕ Semanalmente ⎕ Diariamente
	If you attended these events, on average, how many hours did each time/session last?	Se você esteve em tais eventos, quanto tempo, em média, cada um durou?	If you have been at such events, how long, on average, did each last?	Se você esteve em tais eventos, quanto tempo, em média, cada um durou?
	⎕ 8 hours or more ⎕ 4 hours up to 8 hours ⎕ 1 hour up to 4 hours ⎕ Less than 1 hour	⎕ 8 horas ou mais ⎕ De 4 a 8 horas ⎕ De 1 a 4 horas ⎕ Menos de 1 hora	⎕ 8 hours or more ⎕ 4 to 8 hours ⎕ 1 to 4 hours ⎕ Less than 1hour	⎕ 8 horas ou mais ⎕ De 4 a 8 horas ⎕ De 1 a 4 horas ⎕ Menos de 1 hora
	If you attended these events, how often did you wear earplugs or earmuffs during this activity?	Se você esteve em tais eventos, com que frequência você usou protetor auditivo (tampão de ouvido ou abafador) durante a atividade?	If you have been to such events, how often did you wear hearing protection equipment (earplugs or earmuffs) during the activity?	Se você esteve em tais eventos, com que frequência você usou protetor auditivo (tampão de ouvido ou abafador) durante a atividade?
	⎕ Never ⎕ Sometimes ⎕ Always	⎕ Nunca ⎕ Às vezes ⎕ Sempre	⎕ Never ⎕ Sometimes ⎕ Always	
				⎕ Nunca ⎕ Às vezes ⎕ Sempre
4	How often did you **ride**/operate motorized vehicles such as motorcycles, jet skis, speed boats, **snowmobiles, or four-wheelers**?	Com que frequência você dirigiu/operou veículos motorizados, tais como motocicletas, jet-skis, lanchas ou **quadriciclos**?	How often did you **drive**/operate motorized vehicles, such as motorcycles, jet skis, speed boats **or quads**?	Com que frequência você dirigiu/operou veículos motorizados, tais como motocicletas, jet-skis, lanchas ou quadriciclos**/buggy/Kart**?
	⎕ Never ⎕ Every few months ⎕ Monthly ⎕ Weekly ⎕ Daily	⎕ Nunca ⎕ A cada 2 ou 3 meses ⎕ Mensalmente ⎕ Semanalmente ⎕ Diariamente	⎕ Never ⎕ Every few months ⎕ Monthly ⎕ Weekly ⎕ Daily	⎕ Nunca ⎕ A cada 2 ou 3 meses ⎕ Mensalmente ⎕ Semanalmente ⎕ Diariamente
	If you **rode motorized** vehicles, **on average, how many hours did each time/session last**?	Se você dirigiu veículos motorizados, por quanto tempo, em média, você o usou cada vez?	If you **have driven motor** vehicles, **how long, on average, have you used it each time**?	Se você dirigiu veículos motorizados, por quanto tempo, em média, você o usou cada vez?
	⎕ 8 hours or more ⎕ 4 hours up to 8 hours ⎕ 1 hour up to 4 hours ⎕ Less than 1 hour	⎕ 8 horas ou mais ⎕ De 4 a 8 horas ⎕ De 1 a 4 horas ⎕ Menos de 1 hora	⎕ 8 hours or more ⎕ 4 to 8 hours ⎕ 1 to 4 hours ⎕ Less than 1 hour	⎕ 8 horas ou mais ⎕ De 4 a 8 horas ⎕ De 1 a 4 horas ⎕ Menos de 1 hora
	If you **rode motorized** vehicles, how often **did you wear earplugs or earmuffs during this** activity?	Se você dirigiu veículos motorizados, com que frequência você usou protetor auditivo (tampão de ouvido ou abafador) durante a atividade?	If you **have driven motorized** vehicles, how often **have you used hearing protection equipment (earplugs or earmuffs) during** activity?	Se você dirigiu veículos motorizados, com que frequência você usou protetor auditivo (tampão de ouvido ou abafador) durante a atividade?
	⎕ Never ⎕ Sometimes ⎕ Always	⎕ Nunca ⎕ Às vezes ⎕ Sempre	⎕ Never ⎕ Sometimes ⎕ Always	⎕ Nunca ⎕ Às vezes ⎕ Sempre
5	How often did you ride in or pilot small aircraft/private airplanes?	Com que frequência você voou em, ou pilotou, uma aeronave de pequeno porte/avião particular?	How often did you ride in or pilot small aircraft/private airplanes?	Com que frequência você voou em, ou pilotou, uma aeronave de pequeno porte/avião particular?
	⎕ Never ⎕ Every few months ⎕ Monthly ⎕ Weekly ⎕ Daily	⎕ Nunca ⎕ A cada 2 ou 3 meses ⎕ Mensalmente ⎕ Semanalmente ⎕ Diariamente	⎕ Never ⎕ Every few months ⎕ Monthly ⎕ Weekly ⎕ Daily	⎕ Nunca ⎕ A cada 2 ou 3 meses ⎕ Mensalmente ⎕ Semanalmente ⎕ Diariamente
	If you flew airplanes, on average, how many hours did each time/session last?	Se você voou em aeronaves, quanto tempo, em média, durou cada voo?	If you flew airplanes, on average, how many hours did each time/session last?	Se você voou em aeronaves, quanto tempo, em média, durou cada voo?
	⎕ 8 hours or more ⎕ 4 hours up to 8 hours ⎕ 1 hour up to 4 hours ⎕ Less than 1 hour	⎕ 8 horas ou mais ⎕ De 4 a 8 horas ⎕ De 1 a 4 horas ⎕ Menos de 1 hora	⎕ 8 hours or more ⎕ 4 hours up to 8 hours ⎕ 1 hour up to 4 hours ⎕ Less than 1 hour	⎕ 8 horas ou mais ⎕ De 4 a 8 horas ⎕ De 1 a 4 horas ⎕ Menos de 1 hora
	If you flew airplanes, how often did you wear earplugs or earmuffs during this activity?	Se você voou em aeronaves, com que frequência você usou protetor auditivo (tampão de ouvido ou abafador) durante a atividade?	If you flew airplanes, how often did you wear earplugs or earmuffs during this activity?	Se você voou em aeronaves, com que frequência você usou protetor auditivo (tampão de ouvido ou abafador) durante a atividade?
	⎕ Never ⎕ Sometimes ⎕ Always	⎕ Nunca ⎕ Às vezes ⎕ Sempre	⎕ Never ⎕ Sometimes ⎕ Always	⎕ Nunca ⎕ Às vezes ⎕ Sempre
6	How often **were you around or did you** shoot firearms such as rifles, pistols, shotguns, etc.?	Com que frequência você esteve próximo a, ou disparou, armas de fogo tais como rifles, pistolas, espingardas etc.?	How often **have you been close to, or** shoot firearms such as rifles, pistols, shotguns, etc.?	Com que frequência você esteve próximo a, ou atirou, armas de fogo tais como rifles, pistolas, espingardas etc.?
	⎕ Never ⎕ Every few months ⎕ Monthly ⎕ Weekly ⎕ Daily	⎕ Nunca ⎕ A cada 2 ou 3 meses ⎕ Mensalmente ⎕ Semanalmente ⎕ Diariamente	⎕ Never ⎕ Every few months ⎕ Monthly ⎕ Weekly ⎕ Daily	⎕ Nunca ⎕ A cada 2 ou 3 meses ⎕ Mensalmente ⎕ Semanalmente ⎕ Diariamente
	If you were **around/shot** firearms, **on average, how many shots** did you fire each **time/session**?	Se você esteve próximo a/disparou armas de fogo, quanto disparos, em média, você fez ou presenciou a cada vez?	If you were **close to/ shoot** firearms, **how many shots, on average**, did you fire each **time**?	Se você esteve próximo a/disparou armas de fogo, quanto disparos, em média, você fez ou presenciou a cada vez? _________ tiros de espingarda/rifle por vez. _________ tiros de pistola por vez.
	_________ shotgun/rifle shots **per session** _________ pistol shots **per session**	_________ tiros de espingarda/rifle por vez. _________ tiros de pistola por vez.	_____ shotgun/rifle shots **at a time**. _______ pistol shots **at a time.**	Se você esteve próximo a/disparou armas de fogo, com que frequência você usou protetor auditivo (tampão de ouvido ou abafador) durante os disparos?
	If you were **around/shot** firearms, how often did you wear earplugs or earmuffs **while shooting**?	Se você esteve próximo a/disparou armas de fogo, com que frequência você usou protetor auditivo (tampão de ouvido ou abafador) durante os disparos?	If you were close **to/shot** firearms, how often did you wear earplugs or earmuffs **during the shots**?	⎕ Nunca ⎕ Às vezes ⎕ Sempre
	⎕ Never ⎕ Sometimes ⎕ Always	⎕ Nunca ⎕ Às vezes ⎕ Sempre	⎕ Never ⎕ Sometimes ⎕ Always	
7	How often did you play a musical instrument?	Com que frequência você tocou um instrumento musical?	How often did you play a musical instrument?	Com que frequência você tocou um instrumento musical?
	⎕ Never ⎕ Every few months ⎕ Monthly ⎕ Weekly ⎕ Daily	⎕ Nunca ⎕ A cada 2 ou 3 meses ⎕ Mensalmente ⎕ Semanalmente ⎕ Diariamente	⎕ Never ⎕ Every few months ⎕ Monthly ⎕ Weekly ⎕ Daily	⎕ Nunca ⎕ A cada 2 ou 3 meses ⎕ Mensalmente ⎕ Semanalmente ⎕ Diariamente
	If you played, please tell us what musical instrument: _________________________	Se você tocou, por favor, nos conte qual instrumento: _________________________	If you played, please tell us which instrument: _________________________	Se você tocou, por favor, nos conte qual instrumento:
	If you played a musical instrument, **on average, how many hours did each time/session last**?	Se você tocou um instrumento musical, por quanto tempo, em média, você o tocou cada vez?	If you played a musical instrument **how long, on average, did you play it each time**?	Se você tocou um instrumento musical, por quanto tempo, em média, você o tocou cada vez?
	⎕ 8 hours or more ⎕ 4 hours up to 8 hours ⎕ 1 hour up to 4 hours ⎕ Less than 1 hour	⎕ 8 horas ou mais ⎕ De 4 a 8 horas ⎕ De 1 a 4 horas ⎕ Menos de 1 hora	⎕ 8 hours or more ⎕ 4 to 8 hours ⎕ 1 to 4 hours ⎕ Less than 1 hour	⎕ 8 horas ou mais ⎕ De 4 a 8 horas ⎕ De 1 a 4 horas ⎕ Menos de 1 hora
	If you played a musical instrument, how often did you wear earplugs or earmuffs while playing?	Se você tocou um instrumento musical, com que frequência você usou protetor auditivo (tampão de ouvido ou abafador) enquanto tocava?	If you played a musical instrument, how often did you wear earplugs or earmuffs while playing?	Se você tocou um instrumento musical, com que frequência você usou protetor auditivo (tampão de ouvido ou abafador) enquanto tocava?
	⎕ Never ⎕ Sometimes ⎕ Always	⎕ Nunca ⎕ Às vezes ⎕ Sempre	⎕ Never ⎕ Sometimes ⎕ Always	⎕ Nunca ⎕ Às vezes ⎕ Sempre
8	How often did you listen to music, radio programs, etc. **using personal headsets or earphones**?	Com que frequência você ouviu música, programas de rádio etc., usando fones de ouvido ou headset individuais?	How often did you listen to music, radio programs, etc., **wearing earphones or headsets**?	Com que frequência você ouviu música, programas de rádio etc., usando fones de ouvido ou *headset* individuais?
	⎕ Never ⎕ Every few months ⎕ Monthly ⎕ Weekly ⎕ Daily	⎕ Nunca ⎕ A cada 2 ou 3 meses ⎕ Mensalmente ⎕ Semanalmente ⎕ Diariamente	⎕ Never ⎕ Every few months ⎕ Monthly ⎕ Weekly ⎕ Daily	⎕ Nunca ⎕ A cada 2 ou 3 meses ⎕ Mensalmente ⎕ Semanalmente ⎕ Diariamente
	If you listened through earphones, **on average, how many hours did each time/session last**?	Se você ouviu em fones de ouvido, por quanto tempo, em média, você ouviu cada vez?	If you listened through earphones, **how long, on average, did you use each time**?	Se você ouviu em fones de ouvido, por quanto tempo, em média, você ouviu cada vez?
	⎕ 8 hours or more ⎕ 4 hours up to 8 hours ⎕ 1 hour up to 4 hours ⎕ Less than 1 hour	⎕ 8 horas ou mais ⎕ De 4 a 8 horas ⎕ De 1 a 4 horas ⎕ Menos de 1 hora	⎕ 8 hours or more ⎕ 4 to 8 hours ⎕ 1 to 4 hours ⎕ Less than 1hour	⎕ 8 horas ou mais ⎕ De 4 a 8 horas ⎕ De 1 a 4 horas ⎕ Menos de 1 hora
9	**Other than** music concerts and **headset use** (already covered in questions 3 and 8), how often did you listen to music, radio programs, etc. from audio speakers in a car or at home?	Além de shows de música e uso de headsets (já abordados nas questões 3 e 8), com que frequência você ouviu música, programas de rádio etc. em caixas de som no carro ou em casa?	**In addition to** music concerts and **the use of headsets** (already covered in questions 3 and 8), how often did you listen to music, radio programs, etc. from audio speakers in the car or at home?	Além de *shows* de música e uso de *headsets* (já abordados nas questões 3 e 8), com que frequência você ouviu música, programas de rádio etc. em caixas de som no carro ou em casa?
	⎕ Never ⎕ Every few months ⎕ Monthly ⎕ Weekly ⎕ Daily	⎕ Nunca ⎕ A cada 2 ou 3 meses ⎕ Mensalmente ⎕ Semanalmente ⎕ Diariamente	⎕ Never ⎕ Every few months ⎕ Monthly ⎕ Weekly ⎕ Daily	⎕ Nunca ⎕ A cada 2 ou 3 meses ⎕ Mensalmente ⎕ Semanalmente ⎕ Diariamente
	If you listened via speakers, on average, how many hours did each time/session last?	Se você ouviu em caixas de som, por quanto tempo, em média, você ouviu cada vez?	If you listened via speakers, on average, how many hours did you listen each time?	Se você ouviu em caixas de som, por quanto tempo, em média, você ouviu cada vez?
	⎕ 8 hours or more ⎕ 4 hours up to 8 hours ⎕ 1 hour up to 4 hours ⎕ Less than 1 hour	⎕ 8 horas ou mais ⎕ De 4 a 8 horas ⎕ De 1 a 4 horas ⎕ Menos de 1 hora	⎕ 8 hours or more ⎕ 4 hours up to 8 hours ⎕ 1 hour up to 4 hours ⎕ Less than 1 hour	⎕ 8 horas ou mais ⎕ De 4 a 8 horas ⎕ De 1 a 4 horas ⎕ Menos de 1 hora
10	**Now think back to this past summer**. Over the summer months, did you work **a noisy** paid **job**, such as in construction, farming, **a factory, lawn service, carwash, or other indoor or outdoor job working around loud** equipment or machinery? **By noisy job, we mean sounds so loud that you had** to shout or speak in a **raised voice to be heard at arm’s length.**	Ao longo do último ano, você trabalhou em algum serviço formal ou informal ruidoso, como em construção, fábrica, fazenda, jardinagem, lava-rápido, ou outro serviço em ambiente interno ou externo, próximo a equipamentos ou maquinário ruidosos? O que queremos dizer com serviço (ambiente) ruidoso é que o som seria tão intenso que você teria que gritar ou falar em um tom elevado para que lhe escutassem à distância de um metro.	**Over the past year,** did you work one **or more** noisy paid **jobs**, such as construction, farming, **factory, gardening, car wash, or other service indoors or outdoors close to noisy** equipment or machinery? What we mean by a noisy (ambient) service is that the sound would be so loud that you would have to shout or speak in a **high tone to be heard over a meter away.**	Ao longo do último ano, você trabalhou em algum serviço formal ou informal ruidoso, como em construção, fábrica, jardinagem, lava-rápido, ou outro serviço em ambiente interno ou externo, próximo a equipamentos ou maquinário ruidosos? O que queremos dizer com serviço (ambiente) ruidoso é que o som seria tão intenso que você teria que gritar ou falar em um tom elevado para que lhe escutassem à distância de um metro.
	⎕ Yes ⎕ No (if no, skip to # 11)	⎕ Sim ⎕ Não	⎕ Yes ⎕ No	⎕ Sim ⎕ Não
	If yes, please describe the noisy **job(s**): __________________________________	Se sim, por favor descreva o serviço (ambiente) ruidoso:_____________________________	If yes, please describe the noisy **service (environment)**:	Se sim, por favor descreva o serviço (ambiente) ruidoso:_____________________________
	If you worked **a noisy job**, please **estimate** the number of hours you worked in a typical week:	Se você trabalhou em um serviço (ambiente) ruidoso, por favor dê uma estimativa do número de horas que você trabalhou numa semana normal:	__________________________________	Se você trabalhou em um serviço (ambiente) ruidoso, por favor dê uma estimativa do número de horas que você trabalhou numa semana normal:
	________ **average hours** worked per **typical week during the school year**	________ horas trabalhadas por semana normal neste serviço.	If you worked in a noisy (environment) service, please give an estimate of the number of hours you worked in a typical week:	________ horas trabalhadas por semana normal neste serviço.
	If you worked **a noisy** job **during the school year, did your employer give you earplugs or earmuffs to wear** at work?	Se você trabalhou em um serviço ruidoso, o seu empregador lhe forneceu protetor auditivo (tampão de ouvido ou abafador) para usar no trabalho? ⎕ Sim ⎕ Não	________ **hours** worked per **week normal in this service**	Se você trabalhou em um serviço ruidoso, o seu empregador lhe forneceu protetor auditivo (tampão de ouvido ou abafador) para usar no trabalho? ⎕ Sim ⎕ Não
	⎕ Yes ⎕ No	Com que frequência você usou protetor auditivo (tampão de ouvido ou abafador) quando estava próximo a ruídos intensos nesse serviço?	If you worked **in a noisy** job, **have your employer provided you with earplugs or earmuffs to be used** at work?	Com que frequência você usou protetor auditivo (tampão de ouvido ou abafador) quando estava próximo a ruídos intensos nesse serviço?
	How often did you **wear earplugs or earmuffs when around loud noise at this noisy job**?	⎕ Nunca ⎕ Às vezes ⎕ Sempre	⎕ Yes ⎕ No	⎕ Nunca ⎕ Às vezes ⎕ Sempre
	⎕ Never ⎕ Sometimes ⎕ Always		How often did you **use earplugs or earmuffs when you were close to loud noises in this service**?	
			⎕ Never ⎕ Sometimes ⎕ Always	
11	Other than during the summer, over the past year, did you work one or more noisy paid jobs, such as in construction, farming, a factory, lawn service, carwash, or other indoor or outdoor job working around loud equipment or machinery? By noisy job, we mean sounds so loud that you had to shout or speak in a raised voice to be heard at arm’s length.	-	-	-
	⎕ Yes ⎕ No (if no, you’re done with the survey)			
	If yes, please describe the noisy job(s): __________________________________			
	If you worked a noisy job, please estimate the number of hours you worked in a typical week:			
	________ average hours worked per typical week during the school year			
	If you worked a noisy job during the school year, did your employer give you earplugs or earmuffs to wear at work? ⎕ Yes ⎕ No			
	How often did you wear earplugs or earmuffs when around loud noise at this noisy job?			
	⎕ Never ⎕ Sometimes ⎕ Always			

Na realização da síntese das traduções, optou-se por agrupar as questões 10 e 11, na questão 10, adequando-as à situação brasileira, já que se observou que estas questões, presentes no questionário NEQ original, utilizam como referência a estação do ano, devido às condições climáticas presentes no local de origem do questionário e a tradição local de “trabalhos de verão” (Quadro 1).

Outra modificação ocorreu para a questão número 1 do NEQ-S, que tratava do uso de arma de fogo, que não faz parte da cultura do brasileiro em geral. Ela foi totalmente modificada, utilizando-se a junção de três outras perguntas contidas no “Appendix A” do artigo de referência^(10)^ (Quadro 2).

**Quadro 2:** Perguntas do questionário 1-Minute Noise Screen ou Triagem de Exposição ao Ruído de 1-Minuto durante o processo de tradução e adaptação transcultural

QUESTÕES - NEQ-S	VERSÃO ORIGINAL EM INGLÊS	SÍNTESE DAS TRADUÇÕES PARA O PORTUGUÊS BRASILEIRO	SÍNTESE DAS RETROTRADUÇÕES PARA O INGLÊS	COMITÊ DE ESPECIALISTAS: EQUIVALÊNCIA SEMÂNTICA, IDIOMÁTICA, EXPERENCIAL E LINGUISTICA - VERSÃO PRELIMINAR
Instruções	**DURING THE PAST YEAR (12 months)**	**DURANTE O ÚLTIMO ANO (12 meses):**	**DURING THE PAST YEAR (12 months)**	**DURANTE O ÚLTIMO ANO (12 meses):**
1	How often **were you around or did you** shoot firearms such as rifles, pistols, shotguns, etc.?	Com que frequência você esteve **próximo a, ou disparou, armas de fogo tais como rifles, pistolas, espingarda etc.?**	How often **have you been close to, or** shoot firearms such as rifles, pistols, shotguns, etc.?	Com que frequência você esteve **exposto a sons intensos que fizeram você sentir zumbido, ouvidos tampados, dores ou incômodo nos ouvidos?**
	⎕ Never ⎕ Every few months ⎕ Monthly ⎕ Weekly ⎕ Daily	⎕ Nunca ⎕ A cada 2 ou 3 meses ⎕ Mensalmente ⎕ Semanalmente ⎕ Diariamente	⎕ Never ⎕ Every few months ⎕ Monthly ⎕ Weekly ⎕ Daily	⎕ Nunca ⎕ A cada 2 ou 3 meses ⎕ Mensalmente ⎕ Semanalmente ⎕ Diariamente
2	How often **were you exposed** to loud sounds while working on a **paid job**? **By loud sounds, we mean sounds so loud that you had to shout or speak in a raised voice to be heard at arm’s length.**	Com que frequência você esteve exposto a sons altos enquanto trabalhava em um emprego assalariado? O que queremos dizer com som alto é que você teria que gritar ou falar em um tom elevado para que lhe escutassem à distância de um metro.	How often **have you been** exposed to loud sounds while working on a **wage employment**? **What we mean by loud sound is that you would have to scream or speak in a high tone so that they can hear you at a distance of one meter**.	Com que frequência você esteve exposto a sons intensos enquanto trabalhava em um emprego assalariado? O que queremos dizer com som intenso é que você teria que gritar ou falar em um tom elevado para que lhe escutassem à distância de um metro.
	⎕ Never ⎕ Every few months ⎕ Monthly ⎕ Weekly ⎕ Daily	⎕ Nunca ⎕ A cada 2 ou 3 meses ⎕ Mensalmente ⎕ Semanalmente ⎕ Diariamente	⎕ Never ⎕ Every few months ⎕ Monthly ⎕ Weekly ⎕ Daily	⎕ Nunca ⎕ A cada 2 ou 3 meses ⎕ Mensalmente ⎕ Semanalmente ⎕ Diariamente
3	How ofte**n were you exposed to any other types of loud sounds, such as** power tools, **lawn equipment,** or loud music? **By loud sounds, we mean sounds so loud that you had to shout or speak in a raised voice to be heard at arm’s length.**	Com que frequência você esteve exposto a quaisquer outros tipos de som alto tais como ferramentas elétricas, máquinas de cortar grama, ou música alta? O que queremos dizer com som **alto** é que você teria que gritar ou falar em um tom elevado para que lhe escutassem à distância de um metro.	How often **have you been exposed to any other types of loud sounds such as** power tools, **lawn mowers,** or loud music? **What we want to say with a loud sound is that you would have to shout or scream in a high tone so that listened to you at a distance of one meter.**	Com que frequência você esteve exposto a quaisquer outros tipos de sons intensos tais como ferramentas elétricas, máquinas de cortar grama ou música alta? O que queremos dizer com som intenso é que você teria que gritar ou falar em um tom elevado para que lhe escutassem à distância de um metro.
	⎕ Never ⎕ Every few months ⎕ Monthly ⎕ Weekly ⎕ Daily	⎕ Nunca ⎕ A cada 2 ou 3 meses ⎕ Mensalmente ⎕ Semanalmente ⎕ Diariamente	⎕ Never ⎕ Every few months ⎕ Monthly ⎕ Weekly ⎕ Daily	⎕ Nunca ⎕ A cada 2 ou 3 meses ⎕ Mensalmente ⎕ Semanalmente ⎕ Diariamente

Além disso, houve uma adequação do termo “tampão de ouvido” para “protetor auditivo”, adequando-o a nossa realidade e foi acrescentado o vocabulário, “tampão auditivo de ouvido ou abafador”, como explicação, entre parênteses (Quadro 1).

Após a etapa de retrotradução, foram comparadas as versões retrotraduzidas com as originais, destacando as diferenças, tais como tempos verbais, regras condicionais, falsos cognatos e sinônimos. A partir disso, na etapa de análise pelo comitê de especialistas, houve revisão destas diferenças, sendo realizadas as adaptações necessárias de palavras específicas (Quadros 1 e 2).

Na questão 3 do questionário NEQ, foi acrescentada a palavra “religioso”, por se tratar de um evento que usa sistema amplificado de som e é comumente frequentado pela população brasileira. Na questão 4, houve o acréscimo das palavras “buggy/kart” à categoria de quadriciclos, pois estas são as nomenclaturas mais utilizadas. Já na questão 9, optou-se por usar o termo “caixas de som”, ao invés de “caixas de música”, uma vez que a reprodução pode ocorrer para além de músicas, como por exemplo notícias de rádio e podcasts.

Durante a aplicação do pré-teste, os participantes não apresentaram dificuldades para compreender e responder às perguntas, em ambos os questionários, tanto para as questões cujas formas de respostas eram alternativas ou para as questões 7 e 10, nas quais eram necessárias descrições quanto ao instrumento tocado (quando fosse o caso) ou dos serviços realizados em ambiente ruidoso.

Após a etapa de pré-teste, foi realizada a validação do conteúdo e aparência dos questionários, por meio da apresentação ao comitê de especialistas, dos relatórios contendo os comentários referidos pelo público-alvo, bem como das observações da pesquisadora que acompanhou a aplicação dos instrumentos. Identificou-se a necessidade de pequenas modificações de sentenças e palavras específicas, adaptando-as para equivalência experiencial/semântica, mantendo-se, entretanto, a máxima proximidade com a versão original. As versões finais encontram-se nos Apêndices A e B.

Para testar a confiabilidade do NEQ intra-sujeito, foram avaliadas duas perguntas sobre trabalho ruidoso (Questão 10 do NEQ e Questão 2 do NEQ-S). O teste kappa de concordância entre esses dados combinados foi de 0,550 (p < 0,001), representando concordância moderada.

Já a análise da consistência interna foi feita por meio do coeficiente alfa de Cronbach, que resultou num valor de 0,711, que indica consistência interna substancial.

## DISCUSSÃO

A tradução e a adaptação transcultural de instrumentos estrangeiros vêm crescendo nos últimos anos, permitindo seu uso em outras culturas, garantindo que os dados obtidos expressem o que almejam mensurar e possibilitem a comparação desses dados entre diferentes culturas pela utilização de instrumentos padronizados, bem como minimizem gastos de tempo e dinheiro na produção de novos instrumentos^(14,15)^.

Atualmente, existem diversas estratégias para o processo da tradução e adaptação transcultural, sendo necessária a valorização de todas as etapas, a fim de minimizar erros e perdas das características originais dos instrumentos, que podem ocorrer durante este processo^(14,16)^. Embora não exista um modelo padrão-ouro para a tradução e adaptação transcultural, quatro etapas são essenciais e recomendadas: tradução, retrotradução, revisão por comitê de especialistas, pré-teste, de forma a garantir a validade e confiabilidade do instrumento original^(17)^.

De acordo com a referência metodológica utilizada^(11)^, a qual é amplamente adotada nacional e internacionalmente^(14)^, todas as etapas acima mencionadas foram seguidas. O objetivo foi a equivalência semântica, idiomática, experiencial e conceitual entre o texto original e sua tradução, buscando equacionar as dificuldades causadas por significados múltiplos, além de problemas gramaticais, que pudessem surgir durante o processo, que prejudicassem a compreensão do instrumento pelos membros da população a que se destina.

Durante as etapas de tradução e composição do comitê de especialistas, houve cuidado na escolha dos integrantes, incluindo profissionais com *expertise* na área de Audiologia e Saúde Ocupacional. Em relação a composição amostral, foram incluídos diferentes grupos etários e níveis de escolaridade, expostos e não expostos a ruído ocupacional, garantindo assim a verificação da compreensão dos itens, mas também da aplicabilidade do instrumento em uma amostra diversificada e com um número de participantes maior do que o comumente utilizado na literatura^(11,15,16,18-21)^. No entanto, mesmo com todo cuidado durante a seleção da amostra, pode ter havido a influência de um viés de seleção, que é inerente a qualquer pesquisa que utiliza uma amostra de conveniência.

Após a aplicação do pré-teste, algumas modificações nas questões do NEQ e NEQ-S foram feitas, no que diz respeito tanto a pontuação e contexto, como no processo de tradução literal. Houve a necessidade de duas modificações importantes (questão 10 e 11 do NEQ que foram agrupadas na questão 10; questão 1 do NEQ-S onde foram utilizados outros itens do apêndice do instrumento original para substituição). Estas modificações foram feitas para garantir a compatibilidade das perguntas com a realidade brasileira, permitindo melhor compreensão pela população-alvo. Deve-se mencionar que o comitê de especialistas propôs a junção das questões 10 e 11 do instrumento original para adequação da questão à realidade brasileira (clima e tradição), uma vez que não há costume de execução de empregos de verão. Da mesma forma, a questão 1 do NEQ-S precisou ser modificada, pois esta é uma triagem para indicar risco para PAIR baseada na pontuação de três perguntas. Como não há tradição de caça e uso de armas de fogo pela maioria da população brasileira, o comitê de especialistas optou por utilizar três outras questões que estavam presentes em um instrumento adicional elaborado pelos autores^(10)^ e que investigam sintomas que podem estar presentes após a exposição a ruídos intensos: zumbido, sensação de ouvido tampado, dores ou incômodo nos ouvidos.

A necessidade de modificação de perguntas ou enunciados do instrumento original é relatada por pesquisadores da área, que enfatizam que as diferenças culturais podem exigir tais mudanças, principalmente daqueles que envolvem condições específicas, como citado anteriormente^(22)^ e como observado no presente estudo.

Por tratar-se de um instrumento relativamente novo, publicado em 2017^(10)^, não encontramos outros estudos sobre a tradução e adaptação transcultural do NEQ e NEQ-S para outras línguas e países, o que não permite a comparação com outras versões.

Em relação à confiabilidade do instrumento intra-sujeito, o estudo original^(10)^ utilizou a mesma metodologia, comparando duas questões repetidas sobre a mesma temática (trabalho ruidoso) e obtiveram resultados semelhantes aos nossos (kappa = 0,590 ou concordância moderada).

Na análise da consistência interna do instrumento, o coeficiente alfa de Cronbach foi de 0,711, indicando consistência interna substancial. A consistência interna é uma medida de confiabilidade, que reflete como os itens do questionário estão correlacionados entre si. É importante e desejável que este valor varie entre 0,70 a 0,95^(23)^, como o verificado no presente estudo.

Embora existam outros instrumentos que busquem quantificar a exposição anual ao ruído^(24)^, o NEQ utiliza-se de questões simples baseadas em tarefas, que pode ser usado para estimar a exposição do sujeito ao ruído ocupacional e não ocupacional anual. Além disso, possui a versão triagem (NEQ-S), que possibilita a identificação de indivíduos que estão em risco para PAIR, de forma rápida e de fácil aplicação.

Alguns estudos vêm utilizando o NEQ como forma de caracterizar as doses de exposição ao ruído dos participantes. Dentre eles, pode-se citar: Grinn et al.^(25)^, Spankovich et al.^(26)^, Bernard et al.^(27)^, Athirah e Shahida^(28)^, Powell^(29)^. Eles utilizaram este instrumento para calcular a dose de ruído acumulada anual com base nas atividades auto-relatadas.

Sendo assim, a padronização de novos instrumentos, assim como sua disponibilização, representa uma importante estratégia para o desenvolvimento da ciência, e para o uso por profissionais de saúde, gerando impacto na prática clínica. Além disso, podem representar instrumentos de rastreio para a identificação de ruídos prejudiciais presentes no dia-dia, permitindo o melhor planejamento das intervenções, principalmente na área ocupacional^(10,16,19,20)^.

A próxima etapa do nosso estudo visa aplicar o instrumento traduzido e adaptado, para determinar os valores diagnósticos (sensibilidade, especificidade e acurácia) do NEQ comparando-o com o padrão-ouro (audiometria) para a identificação da PAIR.

## CONCLUSÃO

A tradução e adaptação transcultural foram realizadas de acordo com a metodologia preconizada na literatura nacional e internacional, seguindo as etapas de tradução, retrotradução comitê de especialistas e pré-teste, incluindo-se as equivalências necessárias para a manutenção da validade de face e conteúdo com o instrumento original. A disponibilização dos questionários NEQ e NEQ-S na versão da língua portuguesa brasileira abre novos campos de pesquisa para aprofundamento da quantificação de exposição anual ao ruído.

## Apêndice A Versão final: Questionário de Exposição ao Ruído/*Noise Exposure Questionnaire* - NEQ (Johnson et al.^(10)^) traduzido e adaptado para o português brasileiro.

Questionário de Exposição ao Ruído - *Noise Exposure Questionnaire - NEQ*

**Table:**

INSTRUÇÕES: Por favor, responda as questões a seguir sobre si mesmo, sobre sua audição, e sobre qualquer ruído a que tenha sido exposto ao longo do último ano. Escreva sua resposta na lacuna [______] ou assinale [√ ] a melhor resposta em cada questão.										
Por favor, responda estas perguntas referentes a qualquer som intenso. DURANTE O ÚLTIMO ANO (12 meses):										
1.	Fora do seu trabalho assalariado (considerando o trabalho em domicílio), com que frequência você usou ferramentas elétricas, serras elétricas, ou outras ferramentas de oficina que produzam ruído?									
	□ Nunca	□ A cada 2 ou 3 meses			□ Mensalmente		□ Semanalmente			□ Diariamente
	Se você usou ferramentas elétricas, por quanto tempo, em média, você as usou cada vez?									
	□ 8 horas ou mais		□ De 4 a 8 horas			□ De 1 a 4 horas			□ Menos de 1 hora	
	Se você usou ferramentas elétricas, com que frequência você usou protetor auditivo (tampão de ouvido ou abafador) durante a atividade?									
	□ Nunca			□ Às vezes				□ Sempre		
2.	Fora do seu trabalho assalariado (considerando o trabalho em domicílio), com que frequência você dirigiu veículos pesados ou operou maquinários ruidosos (tais como trator, caminhão, maquinário de fazenda ou de quintal como cortador de grama ou soprador / aspirador de folhas)?									
	□ Nunca	□ A cada 2 ou 3 meses			□ Mensalmente		□ Semanalmente			□ Diariamente
	Se você dirigiu/operou maquinário ruidoso, por quanto tempo, em média, você o usou cada vez?									
	□ 8 horas ou mais		□ De 4 a 8 horas			□ De 1 a 4 horas			□ Menos de 1 hora	
	Se você dirigiu/operou maquinário ruidoso, com que frequência você usou protetor auditivo (tampão de ouvido ou abafador) durante a atividade?									
	□ Nunca			□ Às vezes				□ Sempre		
3.	Com que frequência você foi a corridas de carro, caminhões, eventos esportivos, comerciais, escolares, religiosos, concertos, *shows* de música, festas ou quaisquer outros eventos com caixas de som usadas para fazer anúncios ou tocar músicas?									
	□ Nunca	□ A cada 2 ou 3 meses			□ Mensalmente		□ Semanalmente			□ Diariamente
	Se você esteve em tais eventos, quanto tempo, em média, cada um durou?									
	□ 8 horas ou mais		□ De 4 a 8 horas			□ De 1 a 4 horas			□ Menos de 1 hora	
	Se você esteve em tais eventos, com que frequência você usou protetor auditivo (tampão de ouvido ou abafador) durante a atividade?									
	□ Nunca			□ Às vezes				□ Sempre		
4.	Sem considerar carros, com que frequência você dirigiu, operou veículos motorizados, tais como motocicletas, jet-skis, lanchas, quadriciclos, buggy ou Kart?									
	□ Nunca	□ A cada 2 ou 3 meses			□ Mensalmente		□ Semanalmente			□ Diariamente
	Se você dirigiu veículos motorizados, por quanto tempo, em média, você usou cada vez?									
	□ 8 horas ou mais		□ De 4 a 8 horas			□ De 1 a 4 horas			□ Menos de 1 hora	
	Se você dirigiu veículos motorizados, com que frequência você usou protetor auditivo (tampão de ouvido ou abafador) durante a atividade?									
	□ Nunca			□ Às vezes				□ Sempre		
5.	Com que frequência você voou em, ou pilotou, uma aeronave de pequeno porte ou avião particular?									
	□ Nunca	□ A cada 2 ou 3 meses			□ Mensalmente		□ Semanalmente			□ Diariamente
	Se você voou em aeronaves, quanto tempo, em média, durou cada vôo?									
	□ 8 horas ou mais		□ De 4 a 8 horas			□ De 1 a 4 horas			□ Menos de 1 hora	
	Se você voou em aeronaves, com que frequência você usou protetor auditivo (tampão de ouvido ou abafador) durante a atividade?									
	□ Nunca			□ Às vezes				□ Sempre		
6.	Com que frequência você esteve próximo a, ou atirou, armas de fogo tais como rifles, pistolas, espingardas etc.?									
	□ Nunca	□ A cada 2 ou 3 meses			□ Mensalmente		□ Semanalmente			□ Diariamente
	Se você esteve próximo a / ou disparou armas de fogo, quanto disparos, em média, você fez ou presenciou a cada vez?									
	_________ tiros de espingarda/rifle por vez.					_________ tiros de pistola por vez.				
	Se você esteve próximo a / ou disparou armas de fogo, com que frequência você usou protetor auditivo (tampão de ouvido ou abafador) durante os disparos?									
	□ Nunca			□ Às vezes				□ Sempre		
7.	Com que frequência você tocou um instrumento musical?									
	□ Nunca	□ A cada 2 ou 3 meses			□ Mensalmente		□ Semanalmente			□ Diariamente
	Se você tocou, por favor, nos conte qual instrumento: __________________________________									
	Se você tocou um instrumento musical, por quanto tempo, em média, você o tocou cada vez?									
	□ 8 horas ou mais		□ De 4 a 8 horas			□ De 1 a 4 horas			□ Menos de 1 hora	
	Se você tocou um instrumento musical, com que frequência você usou protetor auditivo (tampão de ouvido ou abafador) enquanto tocava?									
	□ Nunca			□ Às vezes				□ Sempre		
8.	Com que frequência você ouviu música, programas de rádio etc., usando fones de ouvido?									
	□ Nunca	□ A cada 2 ou 3 meses			□ Mensalmente		□ Semanalmente			□ Diariamente
	Se você usou fones de ouvido, por quanto tempo, em média, você utilizou cada vez?									
	□ 8 horas ou mais		□ De 4 a 8 horas			□ De 1 a 4 horas			□ Menos de 1 hora	
9.	Além de *shows* de música e uso de fones de ouvido (já abordados nas questões 3 e 8), com que frequência você ouviu música, programas de rádio etc. em caixas de som no carro ou em casa?									
	□ Nunca	□ A cada 2 ou 3 meses			□ Mensalmente		□ Semanalmente			□ Diariamente
	Por quanto tempo, em média, você utilizou cada vez?									
	□ 8 horas ou mais		□ De 4 a 8 horas			□ De 1 a 4 horas			□ Menos de 1 hora	
10.	Ao longo do último ano, você trabalhou em algum serviço formal ou informal ruidoso (barulhento), como em construção, fábrica, jardinagem, lava-rápido, ou outro serviço em ambiente interno ou externo, próximo a equipamentos ou maquinário ruidosos (barulhentos)? O que queremos dizer com serviço (ambiente) ruidoso (barulhento) é que o som seria tão intenso que você teria que gritar ou falar em um tom elevado para que lhe escutassem à distância de um metro.									
	□ Sim		□ Não							
	Se sim, por favor descreva o serviço (ambiente) ruidoso (barulhento):									
	_________________________________________________________________________________									
	Se você trabalhou em um serviço (ambiente) ruidoso (barulhento), por favor dê uma estimativa do número de horas que você trabalhou numa semana normal:									
	________ horas trabalhadas por semana normal nesse serviço									
	Se você trabalhou em um serviço ruidoso (barulhento), o seu empregador lhe forneceu protetor auditivo (tampão de ouvido ou abafador) para usar no trabalho?									
	□ Sim		□ Não							
	Com que frequência você usou protetor auditivo (tampão de ouvido ou abafador) quando estava próximo a ruídos intensos nesse serviço?									
	□ Nunca			□ Às vezes				□ Sempre		

## Apêndice B Versão final: Triagem de Exposição ao Ruído de 1-Minuto/*1-Minute Noise Screen* (Johnson et al. ^(10)^) traduzido e adaptado para o português brasileiro.

**Table:** Triagem de Exposição ao Ruído de 1-Minuto

AO LONGO DO ÚLTIMO ANO (12 meses):							
1.	Com que frequência você esteve exposto a sons intensos que fizeram você sentir zumbido, ouvidos tampados, dores ou incômodo nos ouvidos? O que queremos dizer com som intenso é que você teria que gritar ou falar em um tom elevado para que lhe escutassem à distância de um metro.						
	**□** Nunca	**□** A cada 2 ou 3 meses		**□** Mensalmente		**□** Semanalmente	**□** Diariamente
2.	Com que frequência você esteve exposto a sons intensos enquanto trabalhava em um emprego assalariado? O que queremos dizer com som intenso é que você teria que gritar ou falar em um tom elevado para que lhe escutassem à distância de um metro.						
	**□** Nunca	**□** A cada 2 ou 3 meses		**□** Mensalmente		**□** Semanalmente	**□** Diariamente
3.	**Fora do seu trabalho assalariado**, com que frequência você esteve exposto a quaisquer outros tipos de sons intensos tais como ferramentas elétricas, música alta ou máquinas de cortar grama? O que queremos dizer com som intenso é que você teria que gritar ou falar em um tom elevado para que lhe escutassem à distância de um metro.						
	**□** Nunca	**□** A cada 2 ou 3 meses		**□** Mensalmente		**□** Semanalmente	**□** Diariamente
					**Pontuação de exposição ao ruído:**		
					**__________**		

Como Calcular Sua Pontuação na Triagem de Exposição ao Ruído de 1-Minuto

Primeiro, atribua os pontos a seguir às suas respostas a cada questão:

**Table:**

	Nunca	A cada 2 ou 3 meses	Mensalmente	Semanalmente	Diariamente
Questão 1.	0	1	2	3	4
Questão 2.	0	1	2	3	4
Questão 3.	0	1	2	3	4

Depois, some as três pontuações individuais para chegar à pontuação total de exposição ao ruído. Coloque essa pontuação total na caixa no canto inferior direito de seu cartão.

Leia, no verso desta folha, a explicação de sua pontuação de exposição ao ruído, bem como sugestões de como administrar o risco de desenvolver perda auditiva induzida por ruído.

Exemplo:

Triagem de Ruído em 1 Minuto: Recomendações

**Table:**

Se sua pontuação de ruído está nesta faixa:	Seu risco é:	Explicação
0 a 4	Risco mais baixo	Com base em sua convivência com ruído ao longo do último ano, o risco de você desenvolver perda auditiva induzida por ruído é relativamente baixo, caso você continue a conviver com níveis semelhantes de ruído no futuro. Porém, se sua exposição se tornar maior, o risco de você desenvolver perda auditiva também aumentará.
		A tolerância de cada pessoa ao ruído é diferente e é difícil prever a susceptibilidade de cada um. Ainda assim, é importante lembrar que o risco aumenta quanto mais intenso for o som, quanto mais tempo você estiver exposto a ele, e quanto mais vezes você estiver exposto. Veja as dicas a seguir para saber como administrar o risco de desenvolver perda auditiva induzida por ruído.
		**Observação especial aos que usam armas de fogo**: Se você usa armas de fogo, seu risco de perda auditiva é alto, mesmo que você use a arma de fogo a cada dois ou três meses e sua pontuação tenha sido baixa na Triagem de Exposição ao Ruído de 1-Minuto. Veja as dicas a seguir para saber o que você pode fazer para administrar esse risco.
5 ou mais	Risco mais elevado	Com base em sua convivência com ruído ao longo do último ano, você corre risco de desenvolver perda auditiva induzida por ruído caso você continue a conviver com níveis semelhantes ou mais elevados de ruído no futuro.
		A tolerância de cada pessoa ao ruído é diferente e é difícil prever a susceptibilidade de cada um. Ainda assim, é importante lembrar que o risco aumenta quanto mais intenso for o som, quanto mais tempo você estiver exposto a ele, e quanto mais vezes você estiver exposto. Veja as dicas a seguir para saber como administrar o risco de desenvolver perda auditiva induzida por ruído.

O que Você Pode Fazer para Administrar o Risco:

**Evite ruídos intensos quando puder:** Pode ser redundante dizer isso, mas evitar ruído intenso é o primeiro passo para conservar sua audição por toda a vida. Lembre-se, quando você precisar gritar para que alguém lhe escute a um metro ou dois de distância, provavelmente o ruído de fundo está em um nível perigoso. Procure produtos mais silenciosos quando for comprar ferramentas ou equipamentos ruidosos, tais como sopradores / aspiradores de folhas e cortadores de grama. E abaixe o volume quando estiver usando equipamentos eletrônicos, tais como celulares e aparelhos de som.

**Use protetor auricular sempre que estiver próximo a ruídos elevados**: Quando você não puder evitar ruídos intensos, use protetores auditivos (tampões de ouvido ou abafadores) que estejam bem ajustados, ainda que essas circunstâncias sejam ocasionais. Protetores auditivos podem ser adquiridos em diversas farmácias e lojas de conveniência, de ferramentas e de esporte. Certifique-se de estar bem treinado para usar e cuidar de seus protetores auriculares, e substituí-los sempre que necessário. O uso adequado e consistente de protetores auriculares pode diminuir seu risco, principalmente se você usa armas de fogo. Nesse caso, a exposição a um único disparo já pode ser suficiente para danificar sua audição se você não estiver usando protetores auriculares.

**Faça testes auditivos regularmente:** Fique de olho na sua audição! Faça testes rotineiros de audição, uma vez por ano se você estiver na categoria de risco mais elevado listada acima, ou se você passar a ter maior exposição a ruídos. Acompanhe os resultados dos seus testes de audição e peça ao seu fonoaudiólogo para comparar seus testes anuais ao mais recente a fim de verificar quaisquer mudanças mais significativas que possam representar uma preocupação.

**Cuide dos seus ouvidos:** Vá ao médico se você perceber problemas tais como mudanças repentinas na audição, dor, plenitude auricular, ou zumbido nos ouvidos.
